# Incidence and Risk Factors for Sport-Related Concussion in Female Youth Athletes Participating in Contact and Collision Invasion Sports: A Systematic Review

**DOI:** 10.1007/s40279-024-02133-x

**Published:** 2024-12-08

**Authors:** Laura Ernst, Jessica Farley, Nikki Milne

**Affiliations:** Faculty of Health Science and Medicine, Bond University, 2 Promethean Way, Robina, Gold Coast, QLD 4226 Australia

## Abstract

**Background:**

The incidence and risk factors for sport-related concussion (SRC) associated with contact and collision invasion sports (CCIS) in female youth are unclear.

**Objectives:**

This systematic review aimed to identify (i) the incidence of and (ii) risk factors for SRC in female youth athletes playing CCIS.

**Methods:**

A systematic search of PubMed, CINAHL, Embase, SPORTDiscus and ProQuest to 8 May, 2024 was conducted. Two reviewers independently screened articles against eligibility criteria and assessed risk of bias (Joanna Briggs Institute Critical Appraisal Tool). Aetiological or intervention studies reporting on SRC incidence or risk factors in female youth athletes (aged 13–19 years and under) participating in CCIS were included. Meta-analyses were conducted to explore SRC incidence and risk factors. For each meta-analysis of SRC incidence rate, sub-group analyses were conducted by sport. Where heterogeneity was above 60% for the meta-analysis of SRC risk/protective factors, sensitivity analyses were conducted.

**Results:**

The search yielded 4509 articles; 66 were included. Sport-related concussion incidence or risk factor data for nine CCIS were extracted. Pooled estimates revealed SRC incidence for female youth athletes in CCIS combined was 0.50/1000 match and practice hours (95% confidence interval [CI] 0.34–0.66). When examined by sport classification, SRC incidence in contact invasion sports was 0.12/1000 match and practice hours (95% CI 0.03–0.21), and in collision invasion sports was 2.08/1000 match and practice hours (95% CI 0.90–3.25). Sub-group analysis by individual sport revealed female youth soccer players had the highest overall SRC incidence rate (0.89/1000 match and practice hours, 95% CI − 0.19 to 1.97) amongst contact sports, and rugby union players had the highest overall SRC incidence rate (4.04/1000 match and practice hours, 95% CI 3.03–5.05) among collision sports. Forty-five studies (68%) reported SRC risk factor data, investigating 12 different potential risk factors. Female youth sustained over 7.5 times the rate of SRC in matches compared with practice (incidence rate ratio 7.52, 95% CI 6.32–8.95, *p* < 0.01) when competing in CCIS; however, considerable heterogeneity existed (*I*^2^ = 84.98%). When exploring potential risk factors, no significant difference was found in SRC rate between female youth lacrosse players wearing versus not wearing headgear (*p* = 0.07). No significant difference was found in SRC rates between female youth athletes competing in younger versus older age groups (incidence rate ratio 0.91, 95% CI 0.52–1.61, *p* = 0.48, *I*^2^ = 0.00%). Insufficient evidence was available to examine remaining risk factors.

**Conclusions:**

This meta-analysis revealed SRC in female youth playing CCIS was higher than previously reported, with SRC rates higher in matches than practice. Soccer had the highest SRC incidence rate for female youth athletes competing in contact invasion sports, whilst rugby union demonstrated the highest SRC incidence rates for collision invasion sports. The results of this review should be interpreted with caution given the lack of representation from some common CCIS codes. Further research is required to examine SRC risk factors in female youth athletes participating in CCIS.

**Clinical Trial Registration:**

OSF Registration: osf.io/s573v.

**Supplementary Information:**

The online version contains supplementary material available at 10.1007/s40279-024-02133-x.

## Key Points

**Table:** 

This review is the first known to critically appraise and summarise the literature available for sport-related concussion (SRC) in female youth athletes participating in contact and collision invasion sports, with meta-analyses.
Female youth sustained 7.5 times the rate of SRC in matches compared with training/practice; however, this result should be interpreted with caution given the considerable level of heterogeneity that existed between studies.
Soccer had the highest incidence rate of SRC for female youth athletes competing in contact sports, whilst rugby union demonstrated the highest SRC incidence rate for collision sports.
Future research should aim to investigate SRC incidence and risk factors among female youth athletes utilising prospective research methods, valid and reliable assessment and screening tools for SRC, a uniform approach to measure exposure, and utilising definitions consistent with the international consensus guidelines. Consistent approaches to these methods would avoid high levels of heterogeneity when grouping results together for a meta-analysis.
Further research is required to establish the significance of the remaining potential risk factors examined in this review (playing position, mechanism of injury, age, session time [period of match or practice], previous history of SRC, access to medical staff, neck strength, protective equipment, years of experience associated with mechanism of injury, league type [female only league vs mixed sex league], and laws/rules/governance), as well as to establish a more comprehensive catalogue of risk factors and their significance for SRC in female youth athletes competing in contact and collision invasion sports.

## Introduction

The female sporting landscape has seen significant investment and expansion in many national and international levels of competition in women’s contact and collision invasion sports (CCIS) over the past decade [1–3]. The flow-on effect of the evolution of this space has resulted in a substantial rise in participation rates of female youth athletes in CCIS [4–7]. Invasion sports are described by Lamas et al. [8] as “a dispute between two teams in a common field, where the main objectives are scoring a goal or point and preventing the opponent from scoring by means of individual, group and team actions” by way of invading the opposition's territory. Many invasion sports include contact and/or collision aspects as rules of the game. Rice et al. [9] define contact invasion sports as sports in which “athletes routinely make contact with each other or inanimate objects”, such as: basketball, field hockey, ice hockey, lacrosse, soccer, handball, ultimate frisbee, camogie, Gaelic Football or water polo. Collision invasion sports are defined by Meehan III et al. [10] as sports in which “purposeful, routine body-to-body collisions are a legal and accepted part of the game”, such as Australian Rules football, American Football, rugby league, rugby union and rugby 7s. With this increased growth in women’s and girls’ CCIS, comes a mounting need for the sports science and medicine fields to better understand and address sport-related concussion (SRC) associated with female participation.

The 2022 Concussion in Sport Group defines SRC as “a traumatic brain injury caused by a direct blow to the head, neck or body resulting in an impulsive force being transmitted to the brain that occurs in sports and exercise-related activities” [11]. Research is constantly evolving with regard to the short-term repercussion of SRC for youth athletes, such as the detrimental impacts on concentration [12], behaviour [13], academic performance [12], balance [14], visuomotor skills [14] and mental health [15]. Further, some studies exploring the impacts of SRC in CCIS, including American Football, soccer and ice hockey, have reported associations between repetitive brain trauma including concussion and the risk of long-term health consequences such as chronic traumatic encephalopathy [16, 17]. Throughout the life stage of youth/adolescence significant musculoskeletal growth and physical development occurs, including: changes in body composition, metabolic and hormonal fluctuations, maturation of organ systems and establishment of nutrient deposits [18]. Definitions for age range-associated data collection and reporting in sport for the life stage of youth vary worldwide [19–22]. For the purpose of this review, youth are defined as those aged 13–19 years and under [23]. Considering the known and potential short-term and long-term impacts of SRC resulting in neurological deficits, cognitive impairments, mental health problems, and in some cases death, the need for further research into SRC is apparent, especially for populations with rapidly developing brains [24]. Therefore, exploring factors leading to minimisation of incidence and potential risk factors associated with head injuries and SRC in youth athletes is a priority [25, 26] to reduce these consequences.

Several studies have examined the incidence of SRC in youth athlete populations [27–29]; however, to date none has examined female youth athletes in isolation. A systematic review published recently by Walshe et al. [30] examined the incidence rates (IRs) of SRC in female contact/collision sports for athletes of all ages. In the review by Walshe et al. [30], a SRC must have been medically diagnosed, the literature must have been published between the years of 2012 and 2021, and studies that estimated exposure were excluded [30]. Given the resourcing disparities faced within female sports compared with male sports, regular access to practitioners such as physiotherapists and team doctors is an ongoing challenge [31]. These barriers negatively impact the ability to collect medically diagnosed SRC data and exact exposure for individual athletes, placing a limitation on reporting SRC IRs that accurately reflect the current landscape of female contact and collision sports [32]. Walshe et al. [30] have recently provided a sound platform to demonstrate a need for future research into the incidence of SRC in female athletes participating in contact and collision sports; however, more specific inclusion parameters are necessary to ascertain an accurate depiction of the SRC rates in female youth athletes.

Along with an increase in research examining the impact of SRC through analysis of incidence, there has been an increase in research exploring SRC awareness [33], identification [34, 35], diagnosis [36], and protocols regarding return to play and injury prevention for those with SRC [33, 37], particularly within youth sport. However, much of this research is dominated by studies examining male youth athletes in contact or collision invasion sports, or combining male and female SRC rates, with little focus on identifying female specific SRC rates and potential risk factors.

Studies across a range of sports, specifically examining youth athletes, have revealed marked sex differences in SRC risk [34], acute management and recovery [38], and outcomes [39] demonstrating differences in mechanisms of injury and SRC incidence in female youth athletes compared with male youth athletes. Several studies have highlighted that female youth athletes are more likely than youth male athletes to sustain a SRC owing to contact with the playing surface, ball or other playing equipment in CCIS [29, 40, 41]. These sex differences in mechanism of SRC highlight the need for aetiological studies to investigate female-specific risk factors for SRC. To appropriately address injury management, prevention and rehabilitation, understanding SRC mechanisms of injury and the range of potential risk factors involved in sustaining an SRC for specific population groups, such as female youth athletes, is critical.

Abrahams et al. [42] conducted a systematic review examining risk factors across both male and female participants of all age groups, competition levels and sports. The most significant findings from this study revealed match play (over practice or training), and previous SRC, are the two leading risk factors for SRC in all sports combined [42]. Whilst a large proportion of the studies examined in this review involved youth athletes, the discussion of the paper did not aim to discern youth specific risk factors associated with SRC compared to adults, nor were sex-specific risk factors examined or any meta-analyses performed [42]. With almost a decade of literature since the Abrahams et al. [42] review, combined with the rapid growth in female participation in CCIS, there is not only a need for an updated review into the risk factors associated with SRC, but there is a need to review the potential risk factors for SRC specific to female youth athlete populations. Therefore, the purpose of this systematic review is to identify and critically appraise and synthesise the available literature to determine (i) the incidence of and (ii) risk factors for SRC in female youth athletes playing contact or collision invasion sports.

## Methods

This systematic review with meta-analyses was conducted in accordance with the Preferred Reporting Items for Systematic Reviews and Meta-analyses (PRISMA) guidelines [43]. The protocol for this systematic review was registered with Open Science Framework on 12 December, 2022 (https://doi.org/10.17605/OSF.IO/S573V).

### Search Strategy and Selection Criteria

Five electronic databases were searched to identify relevant studies, including: PubMed, CINAHL, Embase (Elsevier), SPORTDiscus and ProQuest. All databases were searched up to 8 May, 2024, with no search filters applied. Reference list screening of all systematic reviews identified was completed to detect any articles not originally captured within the search strategy. Theses and dissertations were included in the search strategy to screen for data that met the inclusion criteria. Where such data were identified, a database search was conducted to ascertain whether the data captured within the theses and dissertations had been published in peer-reviewed literature.

A PubMed search strategy was developed in consultation with an experienced Health Science librarian from Bond University. The search strategy combined text words and Medical Subject Heading (MeSH) terms related to concepts of the research question using the CoCoPop (condition, context, population) [44] and PEO (population, exposure, outcome) formats for epidemiological and aetiological questions, respectively [45]. Concepts for the epidemiological aspect included: suspected or medically diagnosed SRC (Co); participation in contact or collision invasion sports (Co); and female youth athletes (Pop). Concepts for the aetiological aspect included: youth athletes participating in contact or collision invasion sports (P); any modifiable or non-modifiable risk factors (E); and self-reported or medically diagnosed SRC (O). All authors contributed to the final revision of the search strategy prior to conducting the search. The final PubMed search strategy was modified to the subject headings and syntax of the other four databases. See the Electronic Supplementary Material (ESM 1) for the full search strategy utilised. The inclusion and exclusion selection criteria for this review are provided in Table 1.

**Table 1:** Inclusion and exclusion criteria

Inclusion criteria	Exclusion criteria
Population must be female youth athletes between the ages of 13 years and < 19 years	Literature reviews, abstracts or posters
Participants must have a suspected (self-reported, reported history or assessed by a concussion assessment tool) or medically diagnosed concussion or mild traumatic brain injury sustained in a sport-related context	Studies that do not distinguish between male and female athlete SRC data
Study designs must be based on the NHMRC levels of evidence guidelines for aetiological and intervention studies [63]	Studies examining limited contact invasion sports such as touch football, touch or flag rugby, or netball because of restricted contact rules
Must investigate a contact or collision invasion sport: basketball, field hockey, ice hockey, lacrosse, soccer, handball, ultimate frisbee, camogie, Gaelic Football, ringette, floorball, water polo, Australian Rules football, American Football, rugby league, rugby union and rugby 7s	Studies that only contain weighted data associated with SRC or athletic exposure, and raw data to calculate unweighted SRC incidence or risk cannot be obtained
Studies must investigate SRC and include a statistical analysis and data associated with SRC incidence or risk, such as IR, ID, AR, RR, OR, IRR, and HR, or the necessary data to calculate a measure of association	Studies that do not distinguish SRC data between contact/collision invasion sports and non-contact sports
Full text must be available	
Must be written in English language	

*AR* absolute risk, *HR* hazard ratio, *ID* incidence density, *IR* incidence rate, *IRR* incidence rate ratio, *NHMRC* National Health and Medical Research Council, *OR* odds ratio, *RR* relative risk, *SRC* sport-related concussion

### Selection and Screening Process

Results from the selected databases were exported to an external citation manager (EndNote X9), by a single author (LE). Duplicate resolution was completed utilising this software. All references were then exported to a web-based software platform, Covidence (Covidence online systematic review platform, Veritas Health Innovation Ltd, Melbourne, VIC, Australia, www.covidence.org) to complete the phases of screening. Two reviewers (NM and LE) independently screened the titles and abstracts against the eligibility criteria utilising Covidence software (Covidence online systematic review platform, Veritas Health Innovation Ltd). Studies that appeared to meet the inclusion criteria from title and abstract were retrieved in full text and were independently examined against the inclusion and exclusion criteria by the same two reviewers. Reviewers then compared studies selected and resolved any discrepancies via a process of joint re-evaluation against eligibility criteria. Any discrepancies that could not be resolved through this process were resolved by a third reviewer (JF) to achieve a final consensus.

### Critical Appraisal of Methodological Quality of Individual Studies

Included studies were critically appraised using the Joanna Briggs Institute Critical Appraisal Tool for prevalence and incidence studies [46]. Critical appraisal of each included study was conducted independently by author (LE) and contributor (LH). The Joanna Briggs Institute Critical Appraisal Tool for prevalence and incidence studies consists of nine items, each scored ‘0’ for ‘No’ or ‘Unclear’, and ‘1’ for ‘Yes’, to give a maximum total score of 9 points [46]. The total scores from each appraisal were converted to percentage scores and then translated into quality ratings, modified from the categories proposed by Kennelly [47] and as used in previous research [48], whereby scores < 45.4% indicated poor methodological quality, 45.4–61% fair quality, and > 61% good quality. Studies with poor methodological quality were excluded from meta-analysis. Cohen’s κ was utilised to determine the level of agreement between appraisers (LE and LH) utilising IBM SPSS Statistics software [49].

### Data Extraction, Synthesis and Analysis

Data extraction was performed by one reviewer (LE), with reviewers (JF and NM) validating the data extracted from the included studies. Data were tabulated in a spreadsheet utilising Microsoft Excel (v365; Microsoft Corporation, Redmond, WA, USA). Data were extracted relating to study authors, publication year, study title, study aim, study design, population and sample size, age, risk factor measured, definition of concussion used, type of contact or collision invasion sport, competition year, competition level, session type (match vs practice) and type of exposure measured. Data pertaining to study methods and results were also extracted, including: number of concussions, match and/or training exposure time; method of SRC diagnosis or reporting (e.g. medical/physiotherapist/sports trainer, self-reported), SRC diagnostic or screening tool used, and statistical outcomes relating to incidence and epidemiological measure of association; including any confidence intervals (CIs) and *p* values reported. A measure of association “quantifies the relationship between an exposure and disease” [50] or health outcome (i.e. SRC), allowing a potential risk factor to be identified. In the investigation of a risk factor, if a protective factor was identified this was reported in the same manner as risk factors. A measure of association can be used to compare two different populations (i.e. female vs male athletes) or groups (i.e. female athletes wearing protective helmets vs female athletes not wearing protective helmets) to identify factors that potentially play aetiological roles for the onset of certain health outcomes (i.e. SRC) [51]. Examples of measures of association include ratios such as: risk ratio (or relative risk [RR]), rate ratio (or incidence rate ratio [IRR]) and odds ratio (OR), noting that “evidence of an association does not imply that the relationship is causal; the association may be artefactual or non-causal as well” [51]. Definitions, equations and interpretations of the statistical measures extracted to examine incidence and measure association are provided in Table 2 [50–53]. Where raw data were reported or available, the authors (LE and JF) independently applied the equations outlined in Table 2 to calculate SRC incidence or risk. The preferred method of calculating IRs was per 1000 hours. This method of reporting provides the most accurate and comparable measure when comparing injury IRs across different sporting codes and is increasingly becoming the preferred reporting method of exposure amongst injury surveillance literature [54–57]. Where the data required to calculate incidence per 1000 hours were not available, the second preferred measure to calculate the IR was per 1000 athletic exposures (AEs) [measured as one athlete’s participation in one match or one practice].

**Table 2:** Definitions, equations, and interpretations of statistics and measures of association utilised within the systematic review

Term	Definition	Equation	Interpretation
Incidence rate (IR)	IR refers to “a measure of frequency with which new cases of illness, injury, or other health condition” [50] occur in a population over a specified period of time (e.g. SRC)	IR = (number of new SRCs/number of AEs) × 1000 Note: different studies utilise different AE measures, methods of AEs used to calculate IR are detailed in ESM 3, 4 and 5	Example A. An IR of 2.5/1000 AEs in female soccer means that on average there are 2.5 SRCs for every 1000 soccer AEs, with an AE defined as 1 athlete participating in 1 practice or competition Example B. An IR of 1.39/1000 hours in female ice hockey means that on average there are 1.39 SRCs for every 1000 hours of ice hockey exposure
Risk ratio (or relative risk, RR)	A RR, also known as a relative risk, compares the risk of a health outcome (e.g. SRC) among an exposed group with the risk of the health outcome among a non-exposed group [52]. Risk refers to the probability that the health outcome will occur	RR = risk of SRC in group of primary exposure interest/risk of SRC in non-exposed comparison group RR = ((*a*/(*a* + *b*))/(*c*/(*c* + *d*)), where *a* is the number of athletes in the primary group of interest who sustained SRC and *b* is the is the number of athletes in the primary group of interest who did not sustain SRC *c* = # in comparison group that sustained SRC *d* = # in comparison group that did not sustain SRC	A RR that is equal to 1.0 indicates that there is no difference in risk between the exposed and unexposed group, revealing that the exposure does not affect the outcome [51]. A RR greater than 1.0 indicates a positive association, or an increased risk for the health outcome occurring in the exposed group (a “risk factor”) [51]. A RR of less than 1.0 indicates a negative association between the exposure and health outcome in the exposed group compared with the unexposed group (a “protective factor”) [51]
Odds ratio (OR)	An OR captures the odds that an event will occur given a particular exposure compared to the odds of the event occurring in a non-exposed group. Odds refers to the probability that an event will happen compared to the probability that the event will not happen [53]	OR = (odds of SRC in the primary group of interest)/(odds of SRC in the comparison group) OR = (*a*/*b*)/(*c*/*d*), where *a* is the is the number of athletes in the primary group of interest who sustained SRC, *b* is the number of athletes in the primary group of interest that did not sustain SRC, *c* is the is the number of athletes in the comparison group who sustained SRC, and *d* is the is the number of athletes in the comparison group who did not sustain SRC	Where the prevalence of a health outcome is low (less than 10%), an OR may approximate the RR or IRR. Otherwise, typically the OR tends to overestimate the RR or IRR [51]. An OR of 1.0 indicates no association, revealing the exposure does not affect the odds of the outcome. An OR greater than 1.0 indicates a positive association, revealing the exposure is associated with higher odds of the outcome (a “risk factor”). An OR less than 1.0 indicates a negative association, revealing the exposure is associated with lower odds of the outcome (a “protective factor”)
Rate ratio (or incidence rate ratio, IRR)	An IRR compares the IRs, person-time rates or mortality rates of an exposed group to an unexposed group to reveal how more (or less) common a particular event happened [51]	IRR = IR of primary group of interest/IR of comparison group	An IRR of 1.0 indicates equal rates in the two groups. An IRR greater than 1.0 indicates an increased risk for the group in the numerator (a “risk factor”). An IRR less than 1.0 indicates a decreased risk for the group in the numerator (a “protective factor”) [52]

*AE* athletic exposure, *SRC* sport-related concussion

Corresponding authors of included studies were contacted via e-mail to request data where additional information was required, such as differentiation of SRC results by sex or sport, raw numbers for total SRCs, total participants or exposure method. Extracted results (or results calculated by authors LE and JF) were grouped together by session type and exposure method to explore SRC incidence and by risk/protective factor to investigate SRC risk. A meta-analysis was conducted when five or more SRC IR results were extracted or calculated of similar exposure method (e.g. per player hours, per AE). To explore risk/protective factors, a meta-analysis was performed when two or more risk results were reported for the same determinant, where appropriate. Meta-analyses were conducted using Stata v.16 [58] applying the DerSimonian and Laird random-effects model. Where CIs were not provided, web-based statistical software MedCalc [59] was used to calculate CI. To assess for heterogeneity between studies, *I*^2^ statistics were calculated. Thresholds for the interpretation of the *I*^2^ statistic were guided by those outlined in Higgins et al. [60]: 0–40%: might not be important, 30–60%: may represent moderate heterogeneity, 50–90%: may represent substantial heterogeneity, and 75–100%: considerable heterogeneity. For each meta-analysis of the SRC IR, sub-group analyses were conducted according to sport classification (e.g. contact sports or collision sports) and individual sport (e.g. soccer, basketball, rugby union). Where heterogeneity was above 60% for the overall analysis of SRC risk/protective factors, sensitivity analyses were conducted through sub-category analyses of CCIS (e.g. projectile object [ball], stick-oriented sports and playing surface [field, court]) to explore whether variations in grouped sub-categories of CCIS were the cause of high levels of heterogeneity. Exclusion or inclusion of studies within each sensitivity analysis for heterogeneity is reported within the results.

Risk of reporting bias was assessed through visual inspection and tabulation of potentially repeated data sets (such as studies that used results from a previous study and combined these with new data, or studies that analysed databases from similar years such as High School RIO [61] or High School NATION-SP [62]). Studies presenting duplicate datasets were excluded from a meta-analysis to limit bias toward results of single study cohorts. To ensure as many years of data collection as possible were covered in the meta-analysis, some High School RIO data had a minor cross-over of datasets utilised. Studies that utilised multiple cohorts including High School RIO data within their overall study results were also included. The level of evidence of each study included in meta-analysis was determined by two authors (LE and JF) using the National Health and Medical Research Council evidence hierarchy [63] (Table 3) with a third reviewer (NM) utilised to achieve a final consensus when required. Level of certainty for each risk factor result was assessed according to adapted definitions from Farley et al. [64] and Abrahams et al. [42] (Tables 4, 5). Where a meta-analysis was not appropriate, a narrative synthesis of key findings was completed.

**Table 3:** National Health and Medical Research Council Evidence Hierarchy: designations of ‘levels of evidence’ according to type of research question (adapted and based on material provided by the National Health and Medical Research Council [representing the Commonwealth of Australia] [63], with permission)

Level	Intervention or screening intervention	Aetiology^a^
I	A systematic review of level II studies	A systematic review of level II studies
II	A randomised controlled trial	A prospective cohort study
III-1	A pseudorandomised controlled trial (i.e. alternate allocation or some other method)	All or none^b^
III-2	A comparative study with concurrent controls: Non-randomised, experimental trial Cohort study Case–control study Interrupted time series with a control group	A retrospective cohort study
III-3	A comparative study without concurrent controls: Historical control study Two or more single-arm study Interrupted time series without a parallel control group	A case–control study
IV	Case series with either post-test or pre-test/post-test outcomes	A cross-sectional study or case series

^a^If it is possible and/or ethical to determine a causal relationship using experimental evidence, then the ‘Intervention’ hierarchy of evidence should be utilised. If it is only possible and/or ethical to determine a causal relationship using observational evidence (i.e. cannot allocate groups to a potential harmful exposure, such as nuclear radiation), then the ‘Aetiology’ hierarchy of evidence should be utilised

^b^All or none of the people with the risk factor(s) experience the outcome; and the data arise from an unselected or representative case series that provides an unbiased representation of the prognostic effect. For example, no smallpox develops in the absence of the specific virus; and clear proof of the causal link has come from the disappearance of smallpox after large-scale vaccination

Sources: hierarchies adapted and modified from: National Health and Medical Research Council [149]; Bandolier [150]; Lijmer et al. [151]; Phillips et al. [152]

**Table 4:** Level of certainty definitions used for the assessment of summary conclusions (adapted from Abrahams et al. [42], with permission, and Farley et al. [64], with permission)

Level of certainty	Definition
High	The associations investigated included evidence from at least two level II studies with a summary conclusion revealing consistent results^a^. The summary conclusion is unlikely to be strongly affected by future studies
Moderate	The associations investigated included evidence from any of the following: (i) only one level II study and level III-1/III-2 studies with a consistent^a^ summary conclusion; (ii) at least two level III-1/III-2 studies with a consistent summary conclusion; or (iii) level II and/or level III-1/III-2 studies with an inconsistent^a^ summary conclusion. As more information becomes available, the summary conclusion could change
Low	The associations investigated included evidence from either: (i) only one level III-1/III-2 study and level IV study with consistent^a^ or inconsistent^a^ results; or (ii) level IV studies only with consistent^a^ or inconsistent^a^ results. More information is needed to be certain of the summary conclusion
Insufficient	The associations investigated included evidence from only one study (regardless of level of evidence). More research is needed to establish an association summary conclusion

^a^Consistent result includes a summary conclusion of ‘clear association’ or ‘no association’. Inconsistent result includes a summary conclusion of ‘inconsistent association’. Study levels pertain to the National Health and Medical Research Council Evidence Hierarchy for aetiology or intervention studies [63], outlined in Table 3

**Table 5:** Summary conclusion definitions to interpret measure of association results between risk factors for female youth athletes competing in contact and collision invasion sports and SRC (adapted from Farley et al. [64], with permission)

Summary conclusion	Criterion
Clear association (consistent result)	≥ 60% of total associations were deemed significant, indicating sufficient evidence to support the significant relationship between a risk factor and SRC
Inconsistent association (inconsistent result)	34–59% of total associations were deemed significant, indicating inconsistent evidence to support the relationship between a risk factor and SRC
No association (consistent result)	≤ 33% of total associations were deemed significant, indicating sufficient evidence to support no relationship between a risk factor and SRC

*SRC* sport-related concussion

## Results

### Literature Search and Selection

**Fig. 1:**
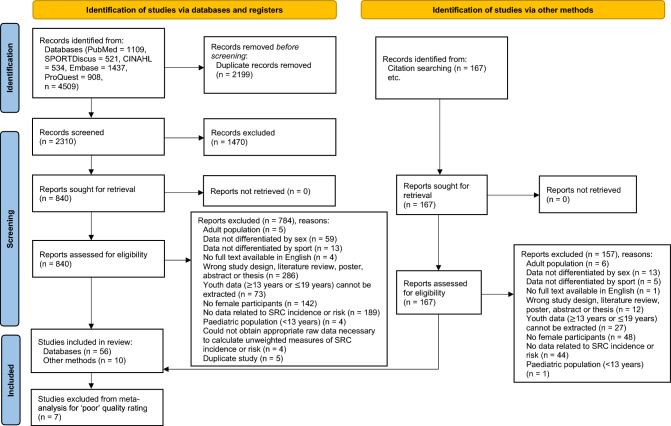
Preferred Reporting Items for Systematic Reviews and Meta-analyses (PRISMA) flow diagram of the selection of studies included in this systematic review and meta-analysis. *SRC* sport-related concussion

The database literature search yielded 4509 articles for review (Fig. 1). One hundred and sixty-seven articles were identified for assessment of eligibility via handsearching of reference lists. The results of the literature search, screening and selection process are summarised in the PRISMA diagram, with 66 articles meeting the inclusion criteria (Fig. 1).

### Methodological Quality of Included Studies

Critical appraisal yielded 11 studies of ‘fair’ quality [65–75] and 48 studies of ‘good’ quality [27, 29, 76–121]. Seven studies were excluded from a meta-analysis due to not meeting the fair-to-good threshold for inclusion in the quantitative analysis [35, 122–127]. Critical appraisal quality percentage scores ranged from 11 to 89% (ESM 2). The mean quality percentage score ± standard deviation of the studies was 66.06 ± 13.94%. There was a moderate level of agreement between the two reviewers (LE and LH), *κ* = 0.72 (*p* < 0.001) [128]. Trends evident from the critical appraisal process revealed lower scoring was mostly due to (i) convenience sampling methods, (ii) failing to detail valid methods used for identification of SRC (i.e. measurement tool) and (iii) failing to compare results across multiple observers when there was more than one data collector.

### Characteristics of Included Studies

Included studies collected female SRC incidence or risk factor data for nine CCIS, which included five contact sports and three collision sports. The representation of the five contact invasion sports amongst the included studies were: soccer (*n* = 37, 56%), basketball (*n* = 25, 38%), lacrosse (*n* = 20, 30%), field hockey (*n* = 11, 17%), ringette (*n* = 1, 2%) and floorball (*n* = 1, 2%) [ESM 2]. The representation of the three collision invasion sports amongst the included studies were: ice hockey (*n* = 9, 14%), rugby union (*n* = 5, 8%) and rugby 7s (*n* = 1, 2%) [ESM 2]. The majority (*n* = 49, 74%) of the included studies investigated high-school athlete populations, with the remaining studies examining youth elite (*n* = 9, 14%), amateur/club or recreational (*n* = 6, 9%), or tournaments (*n* = 2, 3%) [ESM 2]. Types of injury definitions included: medical attention injury, time-loss injury, all complaints injury or no definition was provided. Whilst a utilised definition of SRC was provided in 14 studies (21%), an SRC assessment or screening tool was specified in only 12 studies (18%) [of these 12 studies, 67% also provided a definition of SRC [68, 81, 129–136] utilised in the study] (ESM 2). Procedures for SRC diagnosis and reporting included self-reported SRC, recorded by coaches or school faculty staff, or assessment by medical staff or facility including; certified athletic trainers (AT), physicians, medical doctors, doctors of osteopathic medicine, nurse practitioners, physician’s assistants, physiotherapists, emergency department staff, staff in urgent care facilities, neurologists, orthopaedic physicians, general practitioners, neuropsychologists, emergency medical technicians or other healthcare professionals. An overview of the characteristics of the included studies can be found in the ESM 2.

### IR of SRC

Overall, SRC IR in female athletes participating in CCIS was examined in 50 (76%) of the included studies and subsequently calculated by the authors in an additional 17 studies. Studies reported SRC incidence data utilising varying exposure methods, including: per athlete exposure (defined as one athlete participating in one practice or competition/match) [*n* = 39, 59%], per athlete match or training hours (*n* = 13, 20%), per match hours only (*n* = 7, 11%), per 100 athlete seasons (*n* = 3, 4.5%), per athlete games/matches (*n* = 2, 3%), per 100 athletes per year (*n* = 1, 2%), per 100 team minutes (*n* = 1, 2%) and per athlete days (*n* = 1, 2%). The number of SRCs and IRs sustained by female youth athletes in CCIS were investigated during practice/training (ESM 3), matches (ESM 4) and combined contexts (ESM 5).

**Fig. 2:**
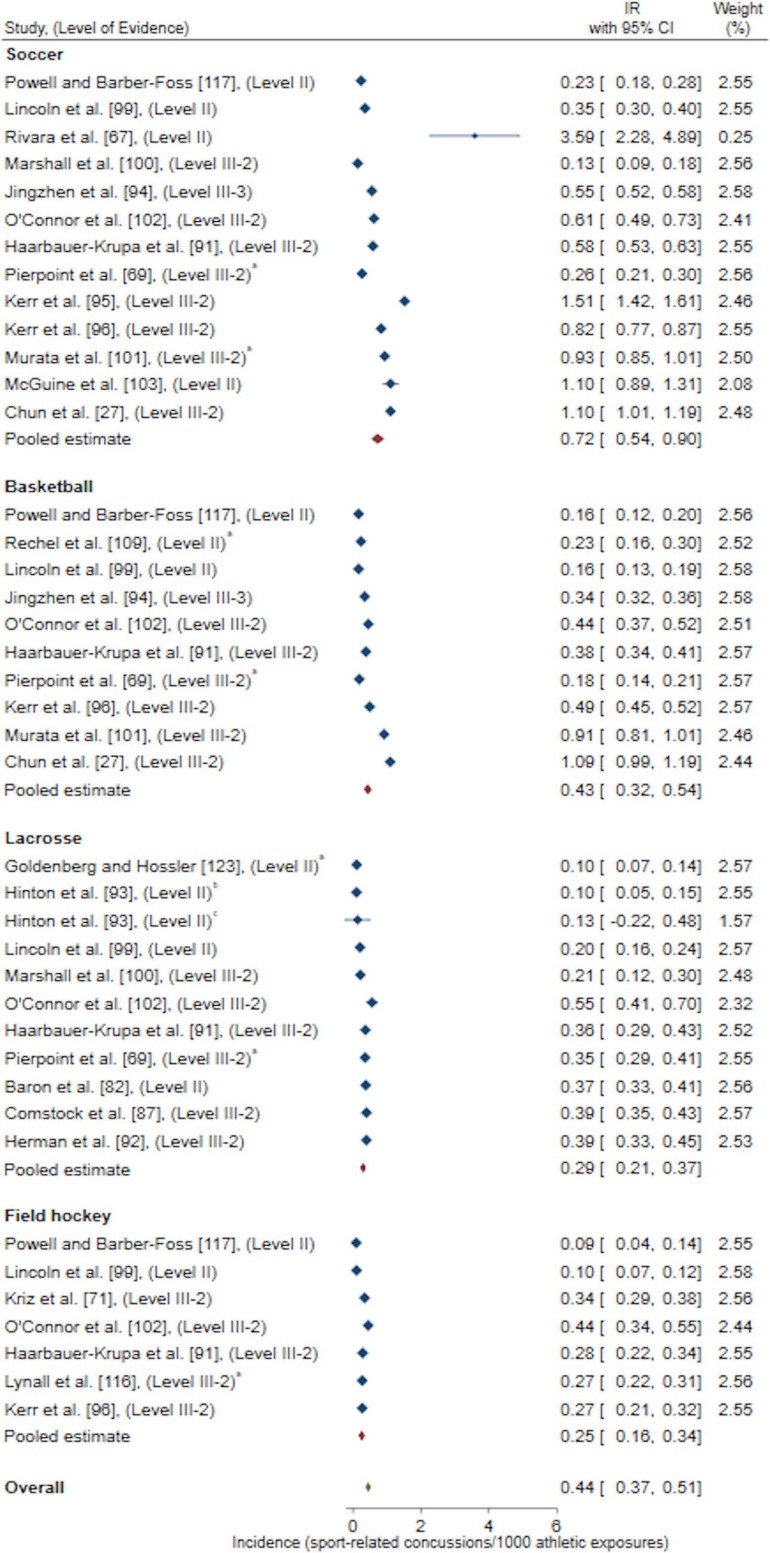
Pooled sport-related concussion incidence rate (IR) for contact invasion sports measured per 1000 athletic exposures, presented in chronological order according to publication year. ^a^Calculated using raw data extracted, ^b^cohort = high school students from Fairfax County Public School System, Virginia, USA, ^c^cohort = female athletes from Elite Summer Lacrosse Camps in Baltimore, MD, USA, *CI* confidence interval, *Level II* prospective cohort study or randomised controlled trial, *Level III-2* retrospective cohort study or a comparative study with concurrent controls, *Level III-3* case–control study or a comparative study without concurrent controls

**Fig. 3:**
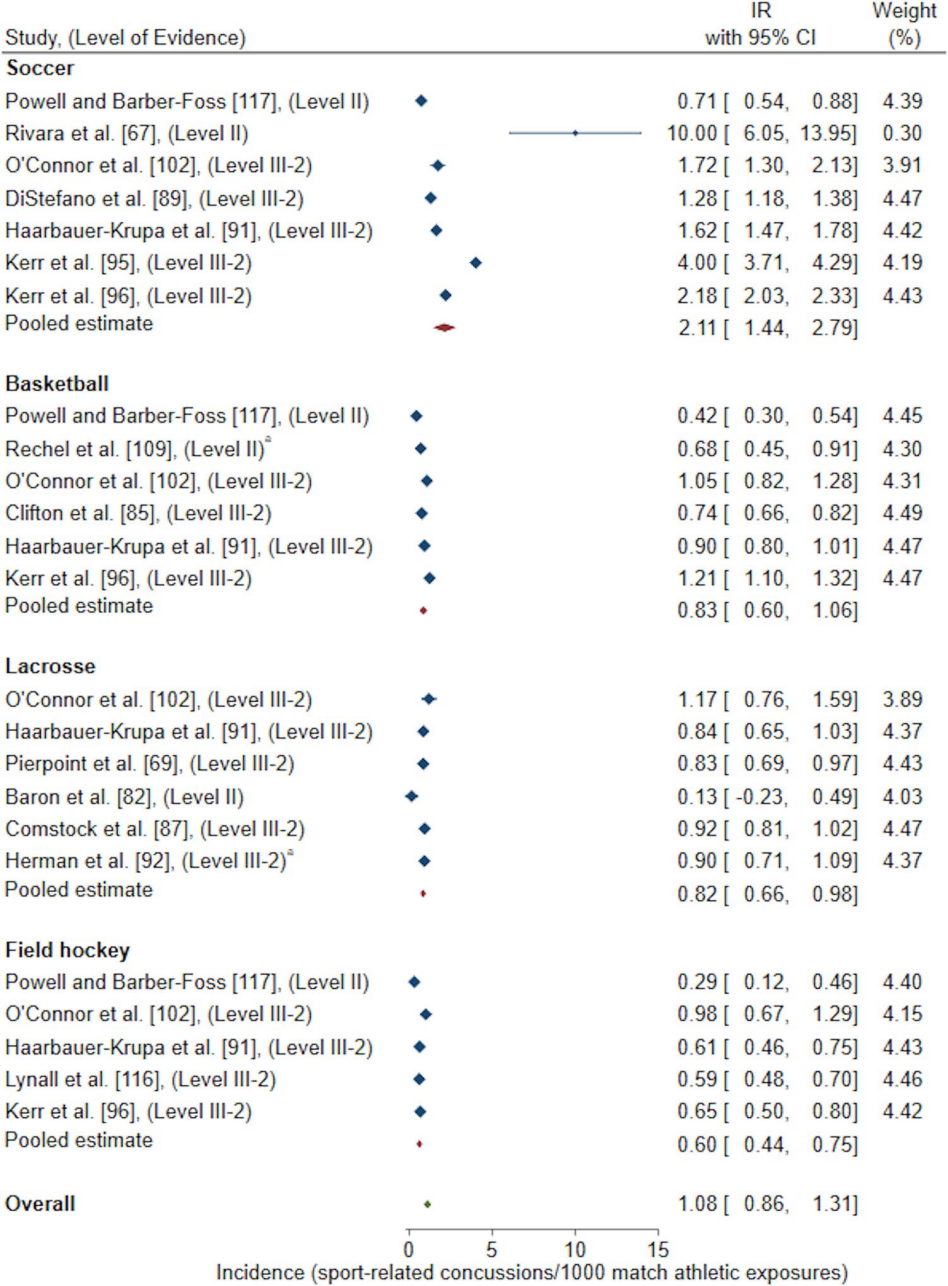
Pooled sport-related concussion incidence rate (IR) for contact invasion sports measured per 1000 match athletic exposures, presented in chronological order according to publication year. ^a^Calculated using raw data extracted, *CI* confidence interval, *Level II* prospective cohort study or a randomised controlled trial, *Level III-2* retrospective cohort study or a comparative study with concurrent controls

**Fig. 4:**
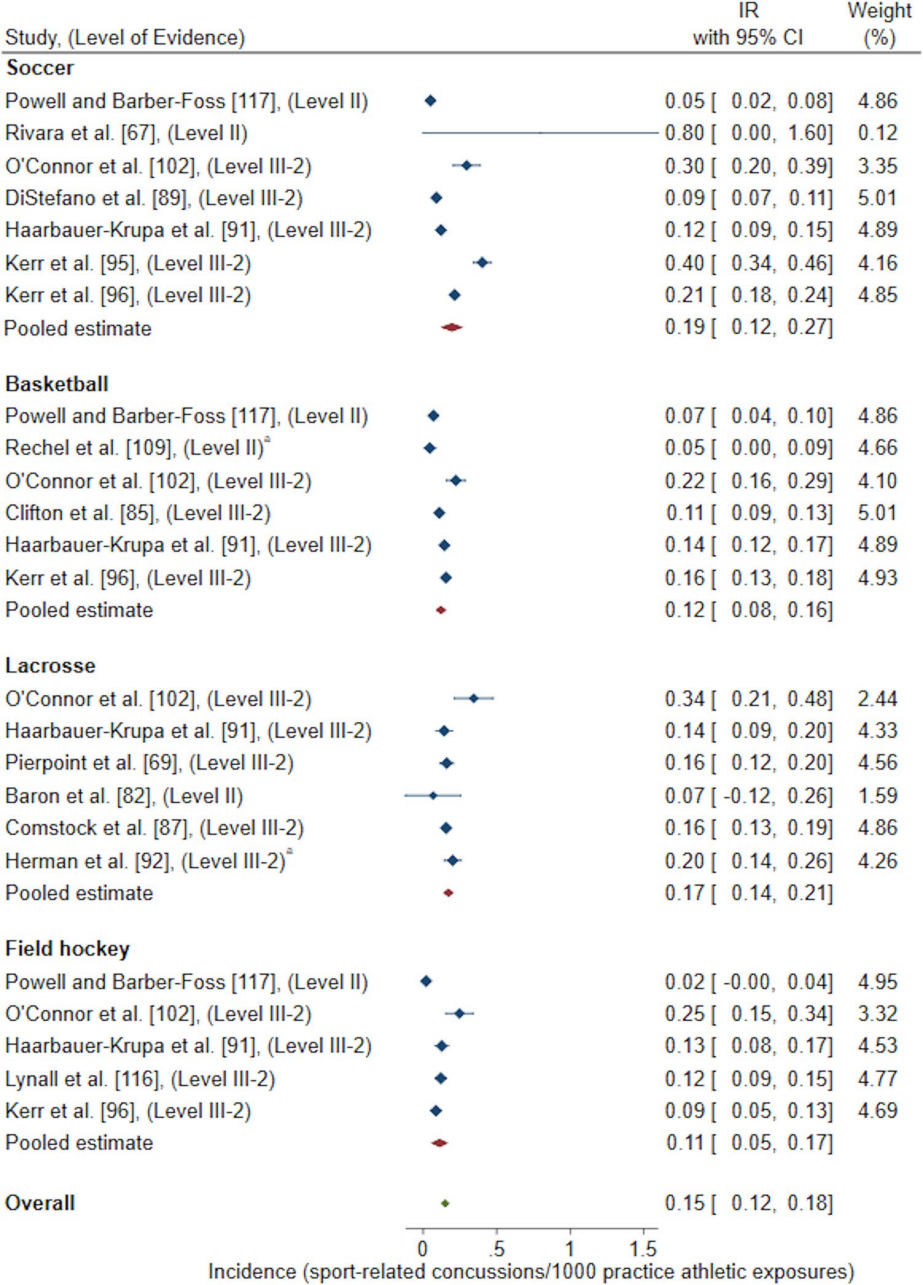
Pooled sport-related concussion incidence rate (IR) for contact invasion sports measured per 1000 practice athletic exposures, presented in chronological order according to publication year. ^a^Calculated using raw data extracted, *CI* confidence interval, *Level II* prospective cohort study or a randomised controlled trial, *Level III-2* retrospective cohort study or a comparative study with concurrent controls

**Fig. 5:**
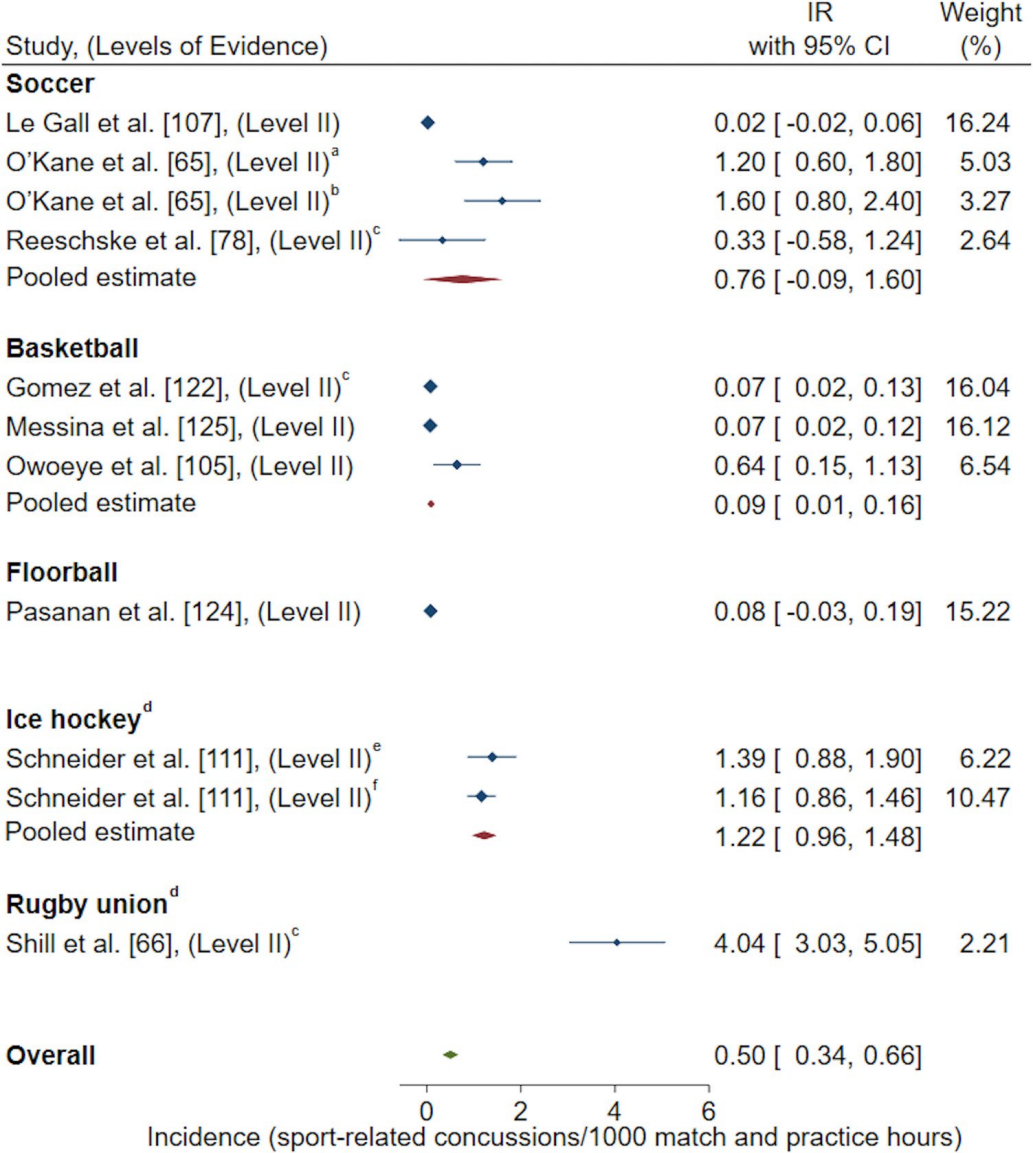
Pooled sport-related concussion incidence rate (IR) for contact and collision invasion sports measured per 1000 match and practice hours combined, presented in chronological order according to publication year. ^a^Cohort = under 14 elite female soccer athletes in the Puget Sound region of Washington State (USA), ^b^cohort = under 15 elite female soccer athletes in the Puget Sound region of Washington State (USA), ^c^calculated using raw data extracted, ^d^collision sports, ^e^cohort = Bantam level elite female ice hockey athletes from youth ice hockey teams from Calgary and Edmonton (Canada), ^f^cohort = Midget level elite female ice hockey athletes from youth ice hockey teams from Calgary and Edmonton (Canada), *CI* confidence interval, *Level II* prospective cohort study or randomised controlled trial

**Fig. 6:**
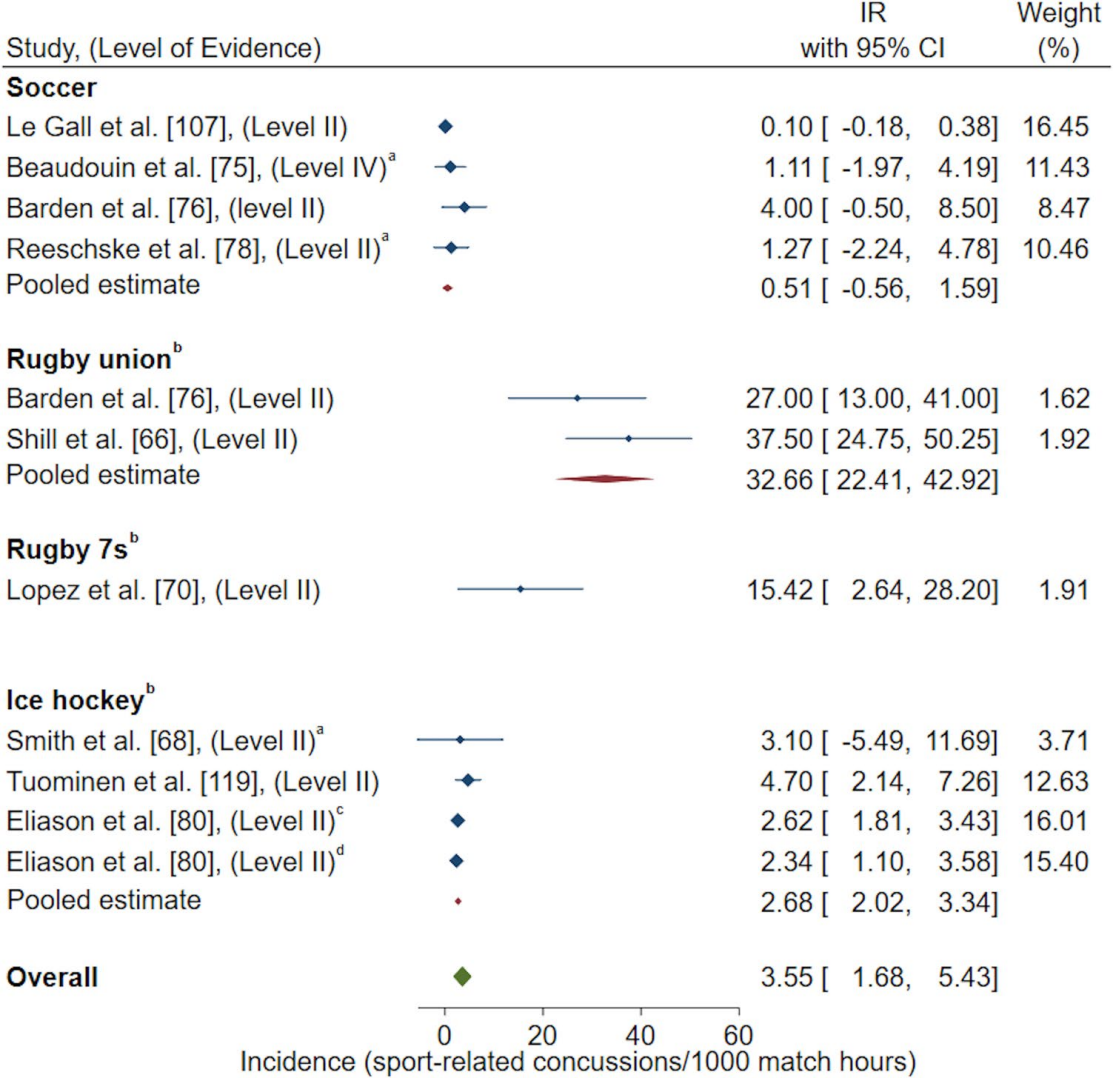
Pooled sport-related concussion incidence rate (IR) for contact and collision invasion sports measured per 1000 match hours, presented in chronological order according to publication year. ^a^Calculated using raw data extracted, ^b^collision sports, ^c^under 15 female ice hockey athletes from youth ice hockey teams across British Columbia and Alberta (Canada), ^d^under 18 female ice hockey athletes from youth ice hockey teams across British Columbia and Alberta (Canada), *CI* confidence interval, *Level II* prospective cohort study or a randomised controlled trial, *Level IV* cross-sectional study or case series or case series with either post-test or pre-test/post-test outcomes

Exposure methods grouped for a meta-analysis resulted in the following reported pooled estimates: IR/1000 AE (one athlete participating in one practice or competition/match) [*n* = 22 studies included in meta-analysis, *n* = 15 studies excluded for duplicate High School RIO data sets, *n* = 1 study excluded as 95% CI could not be calculated, *n* = 1 study excluded as IR was reported for multiple sports combined and could not be differentiated, *n* = 1 study excluded for poor quality rating) [Fig. 2]; IR/1000 match AE (*n* = 14 studies included in meta-analysis, *n* = 5 studies excluded for duplicate High School RIO data sets) [Fig. 3]; IR/1000 practice AE (*n* = 14 studies included in meta-analysis, *n* = 5 studies excluded for duplicate High School RIO data sets) [Fig. 4]; IR/1000 match and practice hours (*n* = 9 studies included in meta-analysis, *n* = 2 studies excluded for duplicate data sets) [Fig. 5]; and IR/1000 match hours (*n* = 9 studies included in meta-analysis, *n* = 1 study excluded for duplicate data set) [Fig. 6]. Sub-group analyses by sport classification resulted in the following reported pooled estimates: IR/1000 match and practice hours for contact sports (*n* = 7); IR/1000 match and practice hours for collision sports (*n* = 2); IR/1000 match hours for contact sports (*n* = 4); and IR/1000 match hours for collision sports (*n* = 6) [ESM 6, 7, 8, 9]. Levels of evidence across the studies included in pooled estimates varied from level II to level IV with the level of evidence noted in each included study for meta-analysis (Figs. 2, 3, 4, 5, 6).

Only contact invasion sports were represented in included studies when examining SRC incidence per AE (ESM 3-5). Pooled estimates revealed the overall SRC IR for female youth athletes in contact invasion sports was 0.44/1000 AE (95% CI 0.37–0.51) (Fig. 2). When examining incidence per match and practice hours, combined pooled estimates of CCIS revealed the overall SRC incidence for female youth athletes was 0.50/1000 match and practice hours (95% CI 0.34–0.66) (Fig. 5). Sub-group analysis for sport classification revealed overall SRC incidence for female youth athletes was 0.12/1000 match and practice hours (95% CI 0.03–0.21) [ESM 6] and 2.08/1000 match and practice hours (95% CI 0.90–3.25) [ESM 7] for contact and collision invasion sports, respectively; demonstrating the overall pooled IR for SRC in collision sports is 17 times higher than the overall pooled IR for SRC in contact sports.

Sub-group analyses by contact invasion sports indicated female youth athletes playing soccer had the highest overall (0.72/1000 AE, 95% CI 0.54–0.90) [Fig. 2], match (2.11/1000 match AE, 95% CI 1.44–2.79) [Fig. 3], and practice (0.19/1000 practice AE, 95% CI 0.12–0.27) [Fig. 4] SRC IRs compared to female youth basketball, lacrosse and field hockey players. A similar result occurred when investigating SRC incidence per overall player hours, where female youth soccer players demonstrated the highest overall SRC IR (0.89/1000 match and practice hours, 95% CI − 0.19 to 1.97) [Fig. 5] amongst contact sports of basketball and floorball. For the examination of SRC IR/1000 match hours only, there was only one contact sport represented; soccer players demonstrated a SRC IR of 0.77/1000 match hours, 95% CI − 0.92 to 2.45 [Fig. 6].

Sub-group analyses by collision invasion sports revealed female youth rugby union players demonstrated the highest overall SRC IR (4.04/1000 match and practice hours, 95% CI 3.03–5.05) followed by ice hockey players (Fig. 5). This finding was also consistent when examining SRC incidence per 1000 match hours, with the highest match SRC IR occurring in rugby union (32.66/1000 match hours, 95% CI 22.41–42.92) followed by rugby 7s and ice hockey (Fig. 6).

### Risk and Protective Factors for SRC

Twenty-five studies (38%) reported SRC risk data, while the authors calculated epidemiological measures of association for 20 studies, investigating a total of 12 different risk factors for SRC in female youth athletes. Non-modifiable risk factors explored included (i) age (ESM 10). Potentially modifiable risk factors explored (i.e. risk factors that may have elements modified via methods such as position rotation, additional education or development of skill sets that may alter likelihood of certain mechanisms of injury, or implementation of protocol or rehabilitation programs for SRC that may result in protective outcomes) included (i) playing position, (ii) mechanism of injury, (iii) years of experience associated with mechanism of injury, (iv) previous history of SRC, (v) session time (period of practice or match), (vi) league type (female only league vs mixed sex league), (vii) session type (match vs practice) and (viii) access to medical staff (ESM 11). Modifiable risk factors explored included (i) neck strength, (ii) protective equipment, and (iii) laws or governance (ESM 12). Session type, age and protective equipment were the three potential risk factors where two or more studies contained results suitable for pooling in meta-analyses.

#### Age (Non-Modifiable)

**Fig. 7:**
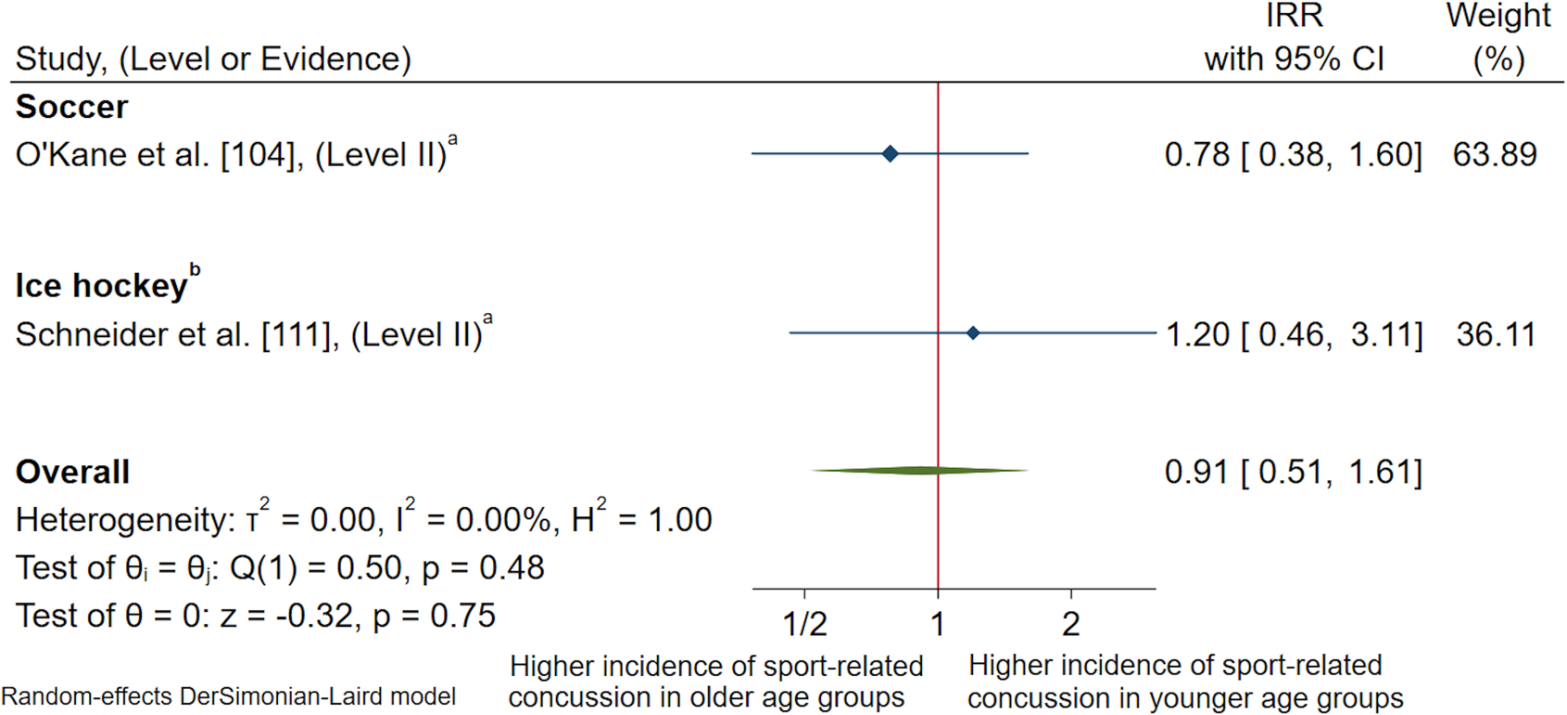
Pooled incidence rate ratios (IRRs) comparing rates of sport-related concussion in younger age groups versus older age groups for contact and collision invasion sports, presented in chronological order according to publication year. ^a^Calculated using raw data extracted, *CI* confidence interval, *Level II* prospective cohort study or randomised controlled trial

Three studies contained the necessary data for authors to calculate a measure of association between age and SRC [77, 104, 111]. All studies demonstrated no significant difference between the rate of SRC between under 15 years age groups compared to over 15 years age groups in the sport of ice hockey, and under 14 years age groups compared to under 15 years age groups in soccer (ESM 10). A meta-analysis of two studies that examined younger age groups versus older age groups in soccer [104] and ice hockey [111] demonstrated no significant difference between the rate of SRC in female youth competing in younger or older age groups (IRR 0.91, 95% CI 0.51–1.61, *p* = 0.48) [Fig. 7]. This result had a moderate level of certainty and a low level of heterogeneity (*I*^2^ = 0.00%).

#### Playing Position (Potentially Modifiable)

Tuominen et al. [119] examined the odds of SRC associated with playing position in ice hockey and found centres had over five times greater odds of sustaining a SRC than defensive players, whilst there was no significant difference seen between wing players compared to defence players or goalies compared to defence players (ESM 11). A study by Lopez Jr et al. [70] provided the necessary data for authors to calculate a measure of association between back and forwards in rugby 7s, and found no significant difference in the rate of SRC between the two positions (ESM 11). These two measures were not appropriate to group together for comparison via meta-analysis (IRR and OR).

#### Mechanism of Injury (Potentially Modifiable)

Five studies (8%) examined or provided the data necessary for the authors to calculate a measure of association between SRC and mechanism of injury. A study by Tuominen et al. [119] examined mechanisms of injury in elite-level ice hockey athletes and found that female athletes had more than five times the odds of sustaining a SRC via checking compared with all other mechanisms. There was no significant difference found when examining checks to the head compared with all other mechanisms, and checking from behind compared to all other mechanisms (ESM 11). Gessel et al. [90] examined the mechanism of injury associated with playing activity in high school basketball athletes and found a significantly greater proportion of SRCs (compared with all other injuries) occurred whilst defending versus any other activity. Kerr et al. [95] provided the necessary data for authors to calculate a measure of association between mechanism of injury and SRC in high-school soccer athletes. The results revealed no significant difference in player-to-player contact compared with all other mechanisms, contact with the ball compared with all other mechanisms, or contact with the ball compared to player-to-player contact (ESM 11). A study by Shill et al. [79] also provided the data necessary for authors to calculate a measure of association between mechanism of injury and SRC in high school rugby union athletes. The results showed no significant difference in tackle related SRCs to the ball carrier versus the tackler in practice, matches, and matches and practice combined. Finally, in a study conducted by Marar et al. [29], the activity being performed at the time of SRC in female high school basketball athletes was examined. The study found that a significantly higher proportion of SRCs (*p* < 0.001) were sustained whilst defending compared with any other activity in basketball matches and practice (incidence proportion ratio [IPR] 2.20, 95% CI 1.60–3.10) [29]. These studies were not suitable to pool for a meta-analysis given the different nature of mechanisms examined and different measures of association (IRR, IPR and OR).

#### Years of Experience Associated with Mechanism of Injury (Potentially Modifiable)

Shill et al. [79] examined the number of years of playing experience and tackle related SRCs in high school rugby union players and found no significant difference in rate of SRC between no years of experience versus 1 year of experience, and no years of experience versus 2 plus years of experience in all tackle-related SRCs (ESM 11). Given there was only one study that examined this risk factor, a meta-analysis of results could not be conducted.

#### Previous History of SRC (Potentially Modifiable)

One study examined subsequent injury patterns following SRC in female high school basketball, field hockey and soccer athletes [108]. No significant association was found between sustaining an initial SRC and sustaining a recurring SRC (ESM 11). Given there was only one study that examined this risk factor, a meta-analysis was not conducted.

#### Session Time (Period of Practice or Match) [Potentially Modifiable]

One study (1.5%) examined the risk of SRC in female high school athletes at various time points of the match compared to practice for soccer, basketball and lacrosse (ESM 11). The risk of SRC appeared to be lowest at the end of soccer match and practices, with the highest risk (RR 6.14, 95% CI 4.34–8.68) occurring when comparing the beginning of a match or practice with the middle of match or practice [88]. In basketball, the risk of sustaining a SRC was also highest at the beginning compared with the middle of a match or practice [RR 5.16, 95% CI 3.73–7.15] [88]. In contrast, in lacrosse there appeared to be no significant difference in SRC risk at the beginning compared with the middle or end, or middle with the end of match or practice sessions. Given there was only one study that examined this risk factor, a meta-analysis of results could not be conducted.

#### League Type (Female-Only League vs Mixed Sex League) [Potentially Modifiable]

A study conducted by Eliason et al. [77] provided the data necessary for authors to calculate a measure of association between the rate of SRC in youth ice hockey athletes participating in female-only leagues versus mixed sex leagues across different levels of league ice hockey in Canada. No significant difference in SRC odds was found between female youth athletes competing in female-only leagues versus female youth athletes competing in mixed sex leagues in youth ice hockey (ESM 11). Given there was only one study that examined this risk factor, a meta-analysis of results could not be conducted.

#### Session Type (Match vs Practice) [Potentially Modifiable]

**Fig. 8:**
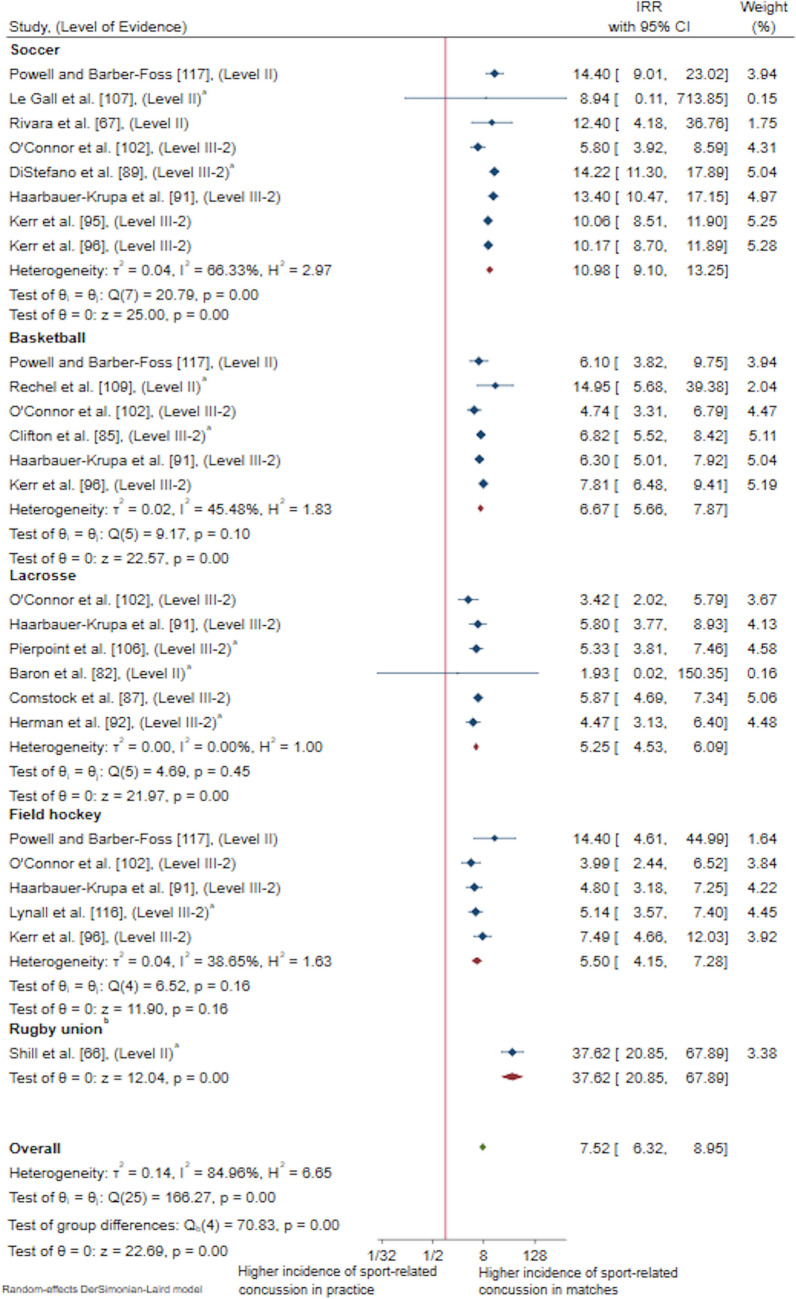
Pooled incidence rate ratios (IRRs) comparing rates of sport-related concussion in match versus practice for contact and collision invasion sports, presented in chronological order according to publication year. ^a^Calculated using raw data extracted, ^b^collision sports, *CI* confidence interval, *Level II* prospective cohort study or randomised controlled trial, *Level III-2* retrospective cohort study or comparative study with concurrent controls

Twenty-one studies (32%) examined or provided the data necessary for the authors to calculate a measure of association between SRC and session type suitable for a meta-analysis, indicated by IRR (*n* = 21). Five studies investigating IRR were subsequently excluded from meta-analysis because of containing duplicate data set years (*n* = 5 High School RIO data duplicates). Meta-analyses revealed that female youth sustained over seven and a half times the rate of SRC in matches compared with practice (IRR 7.52, 95% CI 6.32–8.95, *p* < 0.01) [Fig. 8]. Whilst this result had a high level of certainty, the analysis revealed a substantial heterogeneity (*I*^2^ = 84.96%) and should therefore be interpreted with caution. Sensitivity analysis by projectile object (i.e. ball sports) revealed a higher IR of SRC in matches remained predominant in contact and collision ball sports (IRR 9.49, 95% CI 7.73–11.65, *p* < 0.01, *I*^2^ = 85.20%, *z* = 21.51). To further explore this substantial heterogeneity among contact and collision ball sports, sensitivity analyses by stick sports and playing surface were conducted. Findings revealed SRC IRs continued to remain highest during matches in contact and collision ball sports played with a stick (IRR 5.26, 95% CI 4.61–6.01, *p* < 0.01, *I*^2^ = 11.06%, *z* = 24.50), contact and collision ball sports played on a field (IRR 12.17, 95% CI 9.63–15.39, *p* < 0.01, *I*^2^ = 78.56%, *z* = 20.87), and contact and collision ball sports played on a court (IRR 6.67, 95% CI 5.66–7.87, *p* < 0.01, *I*^2^ = 45.48%, *z* = 22.57).

A sensitivity analysis by sport classification revealed a similar finding with female youth in contact sports sustaining over seven times the rate of SRC in matches compared with practice (IRR 7.11, 95% CI 6.03–8.38], *p* < 0.01, *z* = 23.36); however, heterogeneity remained substantial [*I*^2^ = 82.82%]. This finding remained consistent when examining contact ball sports (IRR 7.11, 95% CI 6.03–8.38, *p* < 0.01, *I*^2^ = 82.82%, *z* = 23.36). Only one study examined risk of session type in collision sports [66]. A full list of all measures of association that were extracted or calculated from the included studies examining session type as a risk factor for SRC in CCIS can be found in the ESM 11.

#### Access to Medical Staff (Potentially Modifiable)

Two studies (3%) investigated how access to medical staff impacted SRC diagnosis in female youth (ESM 11). In a study by Kroshus et al. [98], it was found that having access to an AT made no significant difference to the risk of SRC compared to having no access to an AT in high school soccer athletes (ESM 11). In contrast, in a study by Pierpoint et al. [69], it was found that female youth had eight times the rate of SRC in soccer, and over four times the rate of SRC in basketball when an AT was accessible versus no access to an AT (IRR 8.05, 95% CI 2.00–32.51, *p* < 0.05; and IRR 4.50, 95% CI 1.43–14.16, *p* < 0.05, respectively). These two studies were not suitable to group together for a meta-analysis given the different measures of association utilised (IRR and RR).

#### Neck Strength (Modifiable)

One study examined neck strength measurements between female high school athletes who sustained a SRC compared with those that did not sustain a SRC [73]. Results were reported across the sports of soccer, basketball and lacrosse combined (ESM 12). The study found that for every one-pound (453.6-g) increase in overall neck strength, the odds of sustaining a SRC decreased by 5% (OR 0.95, 95% CI 0.92–0.98) [73]. Given there was only one study that examined this risk factor, a meta-analysis of results could not be conducted.

#### Protective Equipment (Modifiable)

**Fig. 9:**
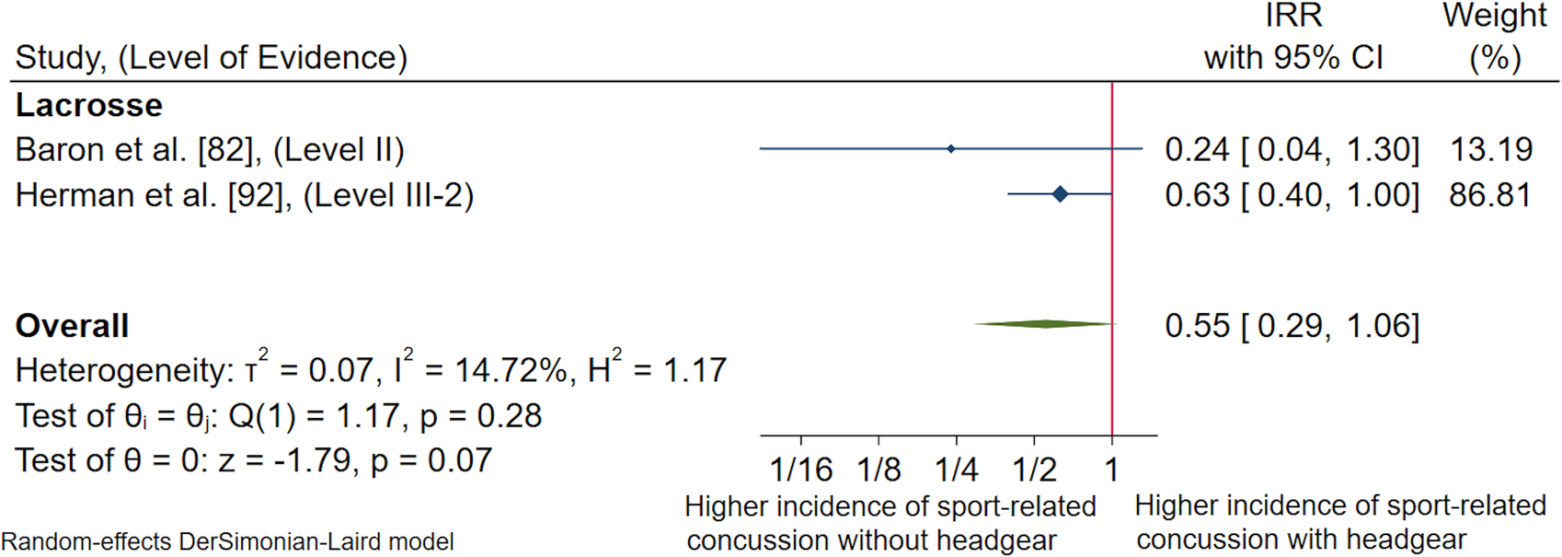
Pooled incidence rate ratios (IRRs) comparing rates of sport-related concussion in female athletes wearing headgear (ASTM F3137 standard for women’s lacrosse) versus athletes not wearing headgear in lacrosse, presented in chronological order according to publication year. *CI* confidence interval, *Level II* prospective cohort study or randomised controlled trial, *Level III-2* retrospective cohort study or comparative study with concurrent controls

Five studies (8%) examined or provided the data necessary for the authors to calculate a measure of association between SRC and the use of protective equipment. Two studies examined the use of protective eyewear in field hockey [71, 72]; however, these studies included duplicate data sets and therefore were not appropriate to group together for a meta-analysis. In the studies by Kriz et al. [71] and Kriz et al. [72], it was found that wearing mandated protective eyewear in female high school competitions made no significant difference to the risk of SRC compared to wearing no protective eyewear (ESM 12). Three remaining studies examined the use of different forms of headgear in soccer [103] and lacrosse [82, 92]. The study by McGuine et al. [103] was subsequently excluded from a meta-analysis due to measures of association not appropriate for comparison. In the study by McGuine et al. [103], the use of soft-form headgear in female high school soccer athletes was examined through a cluster randomised controlled trial. The study found no significant difference in SRC risk between female high school soccer athletes who wore soft-form headgear, and female high school students who did not wear soft-form headgear (ESM 12). A meta-analysis of two studies [82, 92] that examined American Society for Testing and Materials (ASTM) F3137-15 standard lacrosse headgear demonstrated no significant difference between the rate of SRC in female youth with or without the use of headgear (IRR 0.55, 95% CI 0.29–1.06, *p* = 0.07) [Fig. 9]. This result had a moderate level of certainty and a low level of heterogeneity [*I*^2^ = 14.72%]. A full list of all measures of association that were extracted or calculated from the included studies examining the use of protective equipment as a risk or protective factor for SRC in CCIS can be found in the ESM 12.

#### Laws, Rules or Governance (Modifiable)

Three studies (5%) examined the association between SRC laws, rules or governance and the number of SRCs sustained. Two studies examined laws that support the identification of SRC [65, 110], while one study examined rules that aimed to decrease the rate of SRC [68]. Schallmo et al. [110] examined the rate and proportion of SRC in female youth soccer and basketball athletes before and after traumatic brain injury laws in the USA, finding over twice the rate of SRC for female youth soccer players after the law was introduced (IRR 2.52, 95% CI 1.83–3.49, *p* < 0.01) and close to twice the rate of SRC in female youth basketball athletes after the law was introduced (IRR 1.79, 95% CI 1.22–2.62, *p* < 0.01). Conversely, a study by O'Kane et al. [65] that examined the impact of a SRC law (the Zachery Lystedt Law in Washington State, USA) found no significant difference in the risk of SRC before and after the SRC law was implemented in under 14 years of age and under 15 years of age female youth soccer athletes (RR 0.60, 95% CI 0.20–2.10 and RR 1.70, 95% CI 0.50–9.60, respectively). Finally, Smith et al. [68] examined the risk of SRC under intensified fair play rules compared to no intensified fair play rules in female youth ice hockey tournaments and found no significant difference in the risk of SRC with or without intensified fair play rules (RR 0.42, 95% CI 0.02–10.34, *p* = 0.596). A meta-analysis was unable to be conducted with these studies given that the studies examining laws provided different statistical measures of association and therefore data could not be pooled (IRR and RR), and there was only one study that examined rules.

## Discussion and Potential Implications

This systematic review aimed to establish the incidence and risk factors for SRC in female youth athletes participating in CCIS. Pooled estimates revealed the overall incidence of SRC for female youth athletes in contact invasion sports (soccer, basketball, lacrosse and field hockey) combined was 0.44/1000 AE (95% CI 0.37–0.51). A previous review by Pfister et al. [28] examining the incidence of SRC in all youth sports for both male and female individuals combined, demonstrated a pooled estimate of 0.23/1000 AE (95% CI 0.19–0.28). The findings of the present review and meta-analysis suggest the incidence of SRC in female youth may be higher than first described; however, the review by Pfister et al. [28] included a number of collision and non-contact sports, thereby making it difficult to draw accurate comparisons. There are no other known pooled estimates of SRC incidence among female youth sporting populations for comparison.

When examining by sub-group of individual sport, our review revealed the highest rates of SRC in female youth athletes competing in contact sports by session type of match, practice and overall (match and practice combined) when measured /1000 AE were recorded in soccer. When measured /1000 hours (as opposed to /1000 AE), the highest rates of SRC in female youth athletes competing in contact sports also occurred in soccer for matches and overall (matches and practice combined). The overall SRC rate for youth male and female individuals found previously by Pfister et al. [28] for soccer was 0.27/1000 AE (95% CI 0.24–0.30), in contrast to the overall pooled estimate of 0.72/1000 AE (95% CI 0.54–0.90) found for soccer in the present review. These findings demonstrate SRC rates for female youth soccer athletes may be higher than for male youth soccer athletes and may be rising over time, or that surveillance and reporting for SRC is improving. A similar review by Walshe et al. [30] focused on female SRC rates in contact and collision sports across all ages. It reported findings consistent with the present review, revealing that the highest rates of SRC for contact sports were recorded in female soccer athletes. These results may suggest that this trend of high SRC rates is also present in adult levels of competition and warrants further investigation into potential risk factors associated with higher SRC rates in soccer compared with other female contact sports.

The highest rates of SRC in female youth athletes competing in collision sports occurred in rugby union during matches and overall (match and practice combined) when measured per 1000 hours, noting that no studies reported on SRC rates per 1000 AE for collision sports. In a previous review by Kirkwood et al. [137], SRC rates for youth athletes participating in rugby union ranged from 0.2 to 6.9 concussions per 1000 match hours; conversely pooled estimates of SRC for female youth athletes participating in rugby union from included studies in the present review were 32.66/1000 match hours (95% CI 22.41–42.92). Whilst only two studies contributed to pooled estimates in the present review [66, 76], these results remain drastically different. As in soccer, this increased SRC may be due to increasing participation without focused strategies to prevent SRC in female youth or it could simply be due to increased surveillance and reporting; however, the high rate of SRC per 1000 match hours found in this review does highlight the need for further research into SRC in female youth rugby union athletes to better understand these higher than previously published rates. Again, the findings from this review align with the conclusions from the review by Walshe et al. [30], demonstrating that rugby union had the highest rate of SRC for female athletes participating in collision sports. These findings should be interpreted within the context of inherent limitations; however, given both the present review and the review by Walshe et al. [30] did not include studies that reported results for the collision sports of rugby league, Australian Rules football or American Football.

Within this review, IRs were reported for nine different CCIS [27, 29, 35, 65–127]; however, several sports were not represented within the literature including: Australian Rules football, rugby league, Gaelic Football, camogie, American Football, handball, ultimate frisbee and water polo. With high female youth participation levels in sports such as Australian Rules football [138], rugby union [139] and Gaelic Football [140], the findings of the present review demonstrate an important gap within the current body of research regarding SRC IRs in these sports that needs to be addressed [137, 141, 142]. Overall pooled estimates should not necessarily be generalised to all youth female CCIS given the lack of representation across the aforementioned sports, as well as the heavily weighted distribution of study populations within the North American continent. Populations examined were predominantly high-school students based in the USA, with only seven studies containing participants outside of North America [75, 76, 78, 107, 112, 119, 124]. Pooled estimates of incidence may therefore lack generalisability worldwide.

Methods utilised to measure exposure also varied substantially, which resulted in limitations for grouping and comparing all rates within pooled analyses. Increasingly, sporting bodies are shifting towards to use of exposure hours as a measure of athlete exposure, as this provides a more accurate and comparable measure across different sporting codes [54–57]. Whilst AE (measured as one athlete’s participation in one match or one practice) was the most widely adopted measure of exposure utilised across all studies included, it is limited in generalisability across sporting codes given the substantial variation that can exist between length of practice or match time (e.g. basketball matches typically last 40 min in duration, whilst soccer matches typically last 90 min in duration).

Whilst levels of competition were explored, there were only nine studies overall that examined or provided the data necessary for authors to calculate SRC incidence in elite female athlete populations participating in CCIS: ice hockey (*n* = 4) [81, 111, 112, 119], soccer (*n* = 4) [65, 78, 104, 107] and lacrosse (*n* = 1) [93]. With elite national-level and international-level women’s competitions well established in the sports of basketball, soccer, Australian Rules football, rugby union, rugby 7s, ice hockey, rugby league and Gaelic Football, there has been significant investment and growth in the youth talent pathways to these competitions over the past few decades. Despite the significant investment in these sports [1, 2], there is not yet evidence of increased research exploring the incidence and risk of SRC for elite-level female youth athletes participating in these sporting codes.

The present review revealed trends in SRC incidence over time, with all data in IR forest plots presented chronologically according to publishing year, demonstrating an upward trend across most CCIS. This is likely due to several factors such as increased resourcing of medical staff, SRC education, rule modifications, and changes to laws and governance over time but the increased rates may be attributable to other factors, and this requires further monitoring and investigation.

When examining risk factors for SRC in female youth athletes participating in CCIS, the three most prominent risk factors examined were session type, age and the use of protective equipment. Analyses of SRC risk associated with session type in the present review demonstrated that the rate of SRC is significantly higher in matches compared with practice/training and this is consistent with literature examining broader sporting population groups [42]. With increasing awareness of the short-term and long- term consequences of SRC [12–17] and the enforcement of limited contact and tackle practice during training sessions by a variety of sporting codes, it is likely that SRC risk will remain higher in game play than practice in future research.

There were five studies that examined or provided the data necessary for the authors to calculate a measure of association between SRC and the use of protective equipment. A meta-analysis was appropriate for two studies [82, 92] that examined ASTM F3137-15 standard lacrosse headgear. The results demonstrated no difference in SRC rate in female youth with or without the use of ASTM F3137-15 standard lacrosse headgear. The present review revealed limited research across all contact and collision invasion sporting codes to determine any risk or protective effect associated with the use of varying forms of protective equipment in female youth athletes. Further research on the risk or protective effects associated with the use of protective equipment and SRC in female youth athletes is warranted.

Two studies were appropriate to group together for a meta-analysis examining age as a potential risk factor for SRC in female youth athletes competing in CCIS [104, 111]. The results demonstrated no significant difference between the rate of SRC in female youth competing in younger or older age groups. Similarly, the present review found limited research amongst the literature examining age as a potential risk factor for SRC in female youth athletes participating in CCIS. Further research in this area is warranted.

Comparison of IR results between elite and non-elite populations was only available in lacrosse [93]. Variation in exposure methods utilised for the remaining sports examining elite cohorts limited comparisons. Results demonstrate that the elite cohort explored by Hinton et al. [93] had a lower than average SRC rate (0.13/1000 AE, 95% CI 0.00–0.70) than comparable SRC rates in high school cohorts from 13 other studies [29, 87, 91, 92, 96, 99, 100, 102, 106, 113, 114, 123, 126]. This observation should, however, be interpreted with caution given the elite cohort examined in Hinton et al. [93] was participating in a summer camp for aspiring college athletes, where the context of elite competition varies drastically from that of an elite professional league. Specifically, summer camp athletes were attending for further training and game development opportunities, as opposed to elite leagues that play for premiership/championship points and potential promotion or relegation, where they may be likely to receive more contact during game play. No studies examined level of play as a risk factor for SRC, and therefore a meta-analysis could not be performed to explore this question. Further research examining comparisons between elite and non-elite female youth contact and collision invasions sport athletes should be considered.

Some potential risk and protective factors examined should be interpreted within the limitations of the aims of this review. Factors such as access to medical staff, and laws and governance demonstrated increased rates of SRCs for the contact sports of soccer and basketball [69, 110]; however, research has demonstrated that the implementation of SRC identification and reporting methods such as laws and access to medical staff, while demonstrating an increase in initial SRC rates, often leads to a decrease in recurrent SRC rates over time [143]. Remaining risk factors were unable to be examined through meta-analysis due to a limited number of studies examining or providing the necessary data to calculate measures of association, highlighting the need for further studies to explore potential risk factors for SRC within this population group.

### Risk of Bias, Strengths, Limitations and Future Directions

This review is the first known to critically appraise and summarise the literature available for SRC in female youth athletes participating in contact or collision invasion sports, with an accompanying meta-analysis. It is also the first to produce pooled estimates of SRC IRs among female youth athletes competing in CCIS.

Several limitations should be considered when interpreting the findings presented in this systematic review. Whilst a comprehensive search strategy was utilised, studies that were not written in English were excluded from analysis, introducing language bias. Although there were many studies captured in the search strategy, after screening was completed most study populations were located in the continent of North America, which may limit the generalisability of results worldwide. It was also evident following screening that many CCIS were not represented in the literature, including: Australian Rules football, rugby league, Gaelic Football, handball, ultimate frisbee, camogie, water polo and American Football. As such, a lack of representation of these CCIS is an inherent limitation to the conclusions presented in this review. Therefore, results should be interpreted with caution when generalising findings to non-represented CCIS. Measurement bias (i.e. variability in SRC definitions, use of valid and reliable measurement tools for SRC and level of training amongst professionals diagnosing SRC) was also evident across a sizeable number of studies and we believe these variations are likely to be a contributing factor driving the high heterogeneity scores in some meta-analyses. Despite contacting corresponding authors to request data when appropriate (e.g. when original SRC study data did not differentiate by sex, age or type of sport), there were also several raw data sets that were unable to be obtained, which may have impacted the results.

To capture the best available status of SRC incidence and risk among female youth athletes participating in CCIS, methods of reporting such as self-reported SRC were included within the eligibility criteria. This inherently adds a level of bias, however, based on a recent systematic review examining under-reporting of SRC by youth athletes, it is unlikely that the inclusion of studies using self-reported SRC would have impacted the true overall results given the tendency for youth athletes to not disclose SRC symptoms [144]. The decision to include self-reported SRC was also largely influenced by the under-resourcing of trained staff capable of making a diagnosis of SRC present within female youth CCIS [31]. The authors believed that the inclusion of self-reported SRC within these population groups was imperative to provide the most accurate measure of SRC available in this under-researched cohort.

High levels of heterogeneity were observed within the meta-analyses of risk factors. This was expected by the authors given the variations in exposure measurements used, levels of play analysed, SRC diagnostic measures and tools utilised, and the variability amongst contact and collision invasion sporting codes themselves such as length of play, SRC protocols, surfaces, resourcing and equipment. Sensitivity analyses aimed at minimising analyses with known variances, conducted for sub-categories of CCIS demonstrated reduced heterogeneity in most cases. Because of the small number of overall results within each sporting code, further sensitivity analyses were not conducted; however, this should be considered in future research once the body of evidence grows.

With these limitations in mind, future research should aim to investigate SRC incidence and risk factors among female youth athletes utilising prospective research methods, valid and reliable assessment and screening tools for SRC, and utilising definitions consistent with the international consensus guidelines [11]. A uniform approach to measuring exposure should also be adopted, with consensus statements across the CCIS of soccer, rugby union and rugby league all endorsing exposure reporting per 1000 hours [54–57].

Well-established nationwide surveillance programs such as High School RIO [61] and NATION-SP [62] within the USA appear to be a significant factor in the weighting of SRC incidence data amongst the available literature. Countries outside of Northern America should consider similar large-scale surveillance programs within community and school settings to provide more accurate data regarding SRC incidence globally.

## Conclusions

The present systematic review with meta-analysis evaluated the incidence and risk factors for SRC in female youth athletes participating in CCIS. The results demonstrated that SRC incidence in female youth CCIS is higher than the previous literature has reported, and that SRC rates vary across the different types of CCIS examined. Soccer and rugby union appear to have the highest rates of SRC in female youth athletes participating in CCIS, respectively; however, several CCIS were not represented within the literature and the results should therefore be interpreted with caution when generalising findings to non-represented CCIS. The most prominent risk factor for SRC in female youth athletes participating in CCIS was match session type. Whilst high levels of heterogeneity were expected by the authors given the different levels of play analysed, SRC diagnostic measures and tools, and the variability in rules amongst contact and collision invasion sporting codes themselves such as length of play, SRC protocols, surfaces, resourcing and equipment, the findings of this review need to be validated with further research with female youth athletes in a variety of CCIS. The remaining risk factors examined require additional research to ascertain any significance associated with risk or protective effect on SRC. Future research could focus on examining risk and/or protective factors that were not captured within this review, including: genetics, behaviour, playing level, environment (playing surface, weather), anthropometric measures (body mass index), fitness measures, previous injury (other than SRC), menstrual cycle, medication, neurodiverse health conditions (attention-deficit hyperactivity disorder, autism spectrum disorder, developmental coordination disorder), previous symptomology (headaches, dizziness, concertation difficulties), and mental health conditions as these have all been examined in other populations in relation to SRC [42, 145–148]. Prospective research methods, valid and reliable screening and assessment tools for SRC, uniform approaches to measuring athlete exposure and utilising definitions consistent with the international consensus guidelines [57] should be considered. Ongoing research into this field is critical for designing and implementing appropriate injury prevention interventions that align with unique competition environments and requirements in female youth CCIS.

## Supplementary Information

Below is the link to the electronic supplementary material.

Supplementary file1 (PDF 138 KB)

Supplementary file2 (PDF 209 KB)

Supplementary file3 (PDF 55 KB)

Supplementary file4 (PDF 61 KB)

Supplementary file5 (PDF 82 KB)

Supplementary file6 (PDF 382 KB)

Supplementary file7 (PDF 162 KB)

Supplementary file8 (PDF 160 KB)

Supplementary file9 (PDF 335 KB)

Supplementary file10 (PDF 38 KB)

Supplementary file11 (PDF 154 KB)

Supplementary file12 (PDF 66 KB)

## Declarations

### Availability of data and material

The authors confirm that the data supporting the findings of this study are available within the article and its supplementary materials.
