# Supplementary material for: Incidence and Risk Factors for Sport-Related Concussion in Female Youth Athletes Participating in Contact and Collision Invasion Sports: A Systematic Review
Source: Sports Med. 2024 Dec 8;55(2):393–418. doi: 10.1007/s40279-024-02133-x (PMC11947075; doi:10.1007/s40279-024-02133-x)
Supplement: Supplementary file 1 — Supplementary file1 (PDF 138 KB) [file 40279_2024_2133_MOESM1_ESM.pdf]

**Incidence and Risk Factors for Sport-Related Concussion in Female Youth Athletes Participating in Contact and Collision Invasion Sports: A Systematic Review**

**Sports Medicine**

**Laura Ernst[1], Jessica Farley[1], and Nikki Milne[1]**

[1] Faculty of Health Science and Medicine, Bond University, Qld, Australia 4226

* Corresponding Author: Laura Ernst, Email: laura.ernst@student.bond.edu.au

Online Resource 1. Full search strategy utilised for the systematic review from root to 8 May 2024

| **Database** | **Search strategy** | **No of results** |
|---|---|---|
| PubMed | ("youth*"[Title/Abstract] OR "adolescen*"[Title/Abstract] OR "high school"[Title/Abstract] OR "junior*"[Title/Abstract] OR "young"[Title/Abstract] OR "student*"[Title/Abstract] OR "child"[Title/Abstract] OR “teenage*”[Title/Abstract] OR "Adolescent"[MeSH Terms]) AND ("athlete*"[Title/Abstract] OR "player*"[Title/Abstract] OR "Athletes"[MeSH Terms]) AND ("Rugby"[Title/Abstract] OR "Football"[Title/Abstract] OR “collision sport*”[Title/Abstract] OR “contact sport*”[Title/Abstract] OR “tackle*”[Title/Abstract] OR “invasion sport*”[Title/Abstract] OR “Soccer”[Title/Abstract] OR “Hockey”[Title/Abstract] OR “Lacrosse”[Title/Abstract] OR “camogie”[Title/Abstract] OR “Futsal”[Title/Abstract] OR “basketball”[Title/Abstract] OR “handball”[Title/Abstract] OR “ultimate frisbee”[Title/Abstract] OR “water polo”[Title/Abstract] OR "Rugby"[MeSH Terms] OR “Football”[MeSH] OR “Soccer”[MeSH] OR “Hockey”[MeSH] OR “Basketball”[MeSH]) AND ("risk*"[Title/Abstract] OR "determinant*"[Title/Abstract] OR "factor*"[Title/Abstract] OR "influence*"[Title/Abstract] OR "predisposition*"[Title/Abstract] OR "predictor*"[Title/Abstract] OR "relationship*"[Title/Abstract] OR "causa*"[Title/Abstract] OR "association*"[Title/Abstract] OR "mechanism*"[Title/Abstract] OR "etiology"[Title/Abstract] OR "aetiology"[Title/Abstract] OR "odds ratio"[Title/Abstract] OR "hazard ratio"[Title/Abstract] OR "rate ratio"[Title/Abstract] OR "incidence"[Title/Abstract] OR | 1109 |

| | | |
|---|---|---|
| | “probability”[Title/Abstract] OR “epidemiology”[Title/Abstract] OR “prevalence”[Title/Abstract] OR “rate”[Title/Abstract] OR "Risk"[MeSH Terms] OR “Epidemiology”[MeSH] OR “causality”[MeSH]) AND ("concussion*"[Title/Abstract] OR "src"[Title/Abstract] OR "brain injur*"[Title/Abstract] OR "head injur*"[Title/Abstract] OR "mtbi"[Title/Abstract] OR "brain concussion/etiology"[MeSH Terms] OR "youth sports/injuries"[MeSH Terms] OR "adolescent/injuries"[MeSH Terms]) | |
| SportDiscus | ((TI "youth*" OR AB "youth*") OR (TI "adolescen*" OR AB "adolescen*") OR (TI "high school" OR AB "high school") OR (TI "junior*" OR AB "junior*") OR (TI "young" OR AB "young") OR (TI "student*" OR AB "student*") OR (TI "child" OR AB "child") OR (TI "teenage*" OR AB "teenage*") OR DE "Teenagers") AND ((TI "athlete*" OR AB "athlete*") OR (TI "player*" OR AB "player*") OR DE "Athletes") AND ((TI "Rugby" OR AB "Rugby") OR (TI "Football" OR AB "Football") OR (TI "collision sport*" OR AB "collision sport*") OR (TI "contact sport*" OR AB "contact sport*") OR (TI "tackle*" OR AB "tackle*") OR (TI "invasion sport*" OR AB "invasion sport*") OR (TI "soccer" OR AB "soccer") OR (TI "hockey" OR AB "hockey") OR (TI "lacrosse" OR AB "lacrosse") OR (TI "camogie" OR AB "camogie") OR (TI "futsal" OR AB "futsal") OR (TI "basketball" OR AB "basketball") OR (TI "handball" OR AB "handball") OR (TI "ultimate frisbee" OR AB "ultimate frisbee") OR (TI "water polo" OR AB "water polo") OR DE "RUGBY Football" OR DE “FOOTBALL” OR DE “AUSTRALIAN football” OR DE "SOCCER" OR DE "BASKETBALL" OR DE "ULTIMATE (Game)" OR DE "INDOOR soccer” OR DE "CAMOGIE (Game)" OR DE "HOCKEY" OR DE "GAELIC football" OR DE "LACROSSE" OR DE "HANDBALL” OR DE "WATER polo") AND ((TI "risk*" OR AB "risk*") OR (TI "determinant*" OR AB "determinant*") OR (TI "factor*" OR AB "factor*") OR (TI "influence*" OR AB "influence*") OR (TI "predisposition*" OR AB "predisposition*") OR (TI "predictor*" OR AB "predictor*") OR (TI | 521 |

| | | |
|---|---|---|
| | "relationship*" OR AB "relationship*") OR (TI "causa*" OR AB "causa*") OR (TI "association*" OR AB "association*") OR (TI "mechanism*" OR AB "mechanism*") OR (TI "etiology" OR AB "etiology") OR (TI "aetiology" OR AB "aetiology") OR (TI "odds ratio" OR AB "odds ratio") OR (TI "hazard ratio" OR AB "hazard ratio") OR (TI "rate ratio" OR AB "rate ratio") OR (TI "incidence" OR AB "incidence") OR (TI "probability" OR AB "probability") OR (TI “epidemiology” OR AB “epidemiology”) OR (TI “prevalence” OR AB “prevalence”) OR (TI “rate” OR AB “rate”) OR DE "ETIOLOGY of diseases” OR DE "EPIDEMIOLOGY") AND ((TI "src" OR AB "src") OR (TI "concussion*" OR AB "concussion*") OR (TI "brain injur*" OR AB "brain injur*") OR (TI "head injur*" OR AB "head injur*") OR (TI "mtbi" OR AB "mtbi") OR DE "brain concussion") | |
| CINAHL | ((TI youth* OR AB youth*) OR (TI adolescen* OR AB adolescen*) OR (TI "high school" OR AB "high school") OR (TI junior* OR AB junior*) OR (TI young OR AB young) OR (TI student* OR AB student*) OR (TI child OR AB child) OR (TI teenage* OR AB teenage*) OR (MH Adolescent+)) AND ((TI athlete* OR AB athlete*) OR (TI player* OR AB player*) OR (MH Athletes+)) AND ((TI Rugby OR AB Rugby) OR (TI Football OR AB Football) OR (TI "collision sport*" OR AB "collision sport*") OR (TI "contact sport*" OR AB "contact sport*") OR (TI tackle* OR AB tackle*) OR (TI "invasion sport*" OR AB "invasion sport*") OR (TI soccer OR AB soccer) OR (TI hockey OR AB hockey) OR (TI lacrosse OR AB lacrosse) OR (TI camogie OR AB camogie) OR (TI futsal OR AB futsal) OR (TI basketball OR AB basketball) OR (TI handball OR AB handball) OR (TI “ultimate frisbee” OR AB “ultimate frisbee”) OR (TI “water polo" OR AB "water polo") OR (MH “Rugby”+) OR (MH “Australian Football”+) OR (MH “Football”+) OR (MH “Soccer”+) OR (MH “Basketball”+) OR (MH “Hockey”+) OR (MH “Handball”+)) AND ((TI risk* OR AB risk*) OR (TI determinant* OR AB determinant*) OR (TI factor* OR AB factor*) OR (TI influence* OR AB influence*) OR (TI predisposition* OR AB predisposition*) OR (TI | 534 |

| | | |
|---|---|---|
| | predictor* OR AB predictor*) OR (TI relationship* OR AB relationship*) OR (TI causa* OR AB causa*) OR (TI association* OR AB association*) OR (TI mechanism* OR AB mechanism*) OR (TI etiology OR AB etiology) OR (TI aetiology OR AB aetiology) OR (TI "odds ratio" OR AB "odds ratio") OR (TI "hazard ratio" OR AB "hazard ratio") OR (TI "rate ratio" OR AB "rate ratio") OR (TI incidence OR AB incidence) OR (TI prevalence OR AB prevalence) OR (TI epidemiology OR AB epidemiology) OR (TI rate OR AB rate) OR (TI probability OR AB probability) OR (MH “attributable risk”+) OR (MH “epidemiology”+)) AND ((TI src OR AB src) OR (TI concussion* OR AB concussion*) OR (TI "brain injur*" OR AB "brain injur*") OR (TI "head injur*" OR AB "head injur*") OR (TI mtbi OR AB mtbi) OR (MH "brain concussion"+)) | |
| Embase | (youth*:ti,ab OR adolescen*:ti,ab OR 'high school':ti,ab OR junior*:ti,ab OR young:ti,ab OR student*:ti,ab OR child:ti,ab OR teenage*:ti,ab OR Adolescent/exp) AND (athlete*:ti,ab OR player*:ti,ab OR Athletes/exp) AND (Rugby:ti,ab OR Football:ti,ab OR ‘Collision sport*’:ti,ab OR ‘contact sport*’:ti,ab OR tackle*:ti,ab OR ‘invasion sport*’:ti,ab OR soccer:ti,ab OR hockey:ti,ab OR lacrosse:ti,ab OR camogie:ti,ab OR futsal:ti,ab OR basketball:ti,ab OR handball:ti,ab OR ‘ultimate frisbee’:ti,ab OR ‘water polo’:ti,ab OR Rugby/exp OR Football/exp OR Soccer/exp OR Basketball/exp OR Hockey/exp OR ‘ice hockey’/exp OR lacrosse/exp OR ‘water polo’/exp OR futsal/exp OR handball/exp) AND (risk*:ti,ab OR determinant*:ti,ab OR factor*:ti,ab OR influence*:ti,ab OR predisposition*:ti,ab OR predictor*:ti,ab OR relationship*:ti,ab OR causa*:ti,ab OR association*:ti,ab OR mechanism*:ti,ab OR etiology:ti,ab OR aetiology:ti,ab OR 'odds ratio':ti,ab OR 'hazard ratio':ti,ab OR 'rate ratio':ti,ab OR incidence:ti,ab OR probability:ti,ab OR epidemiology:ti,ab OR rate:ti,ab OR prevalence:ti,ab OR Risk/exp OR Epidemiology/exp OR incidence/exp OR prevalence/exp) AND (src:ti,ab OR concussion*:ti,ab OR 'brain injur*':ti,ab OR 'head injur*':ti,ab OR mtbi:ti,ab OR 'brain concussion'/exp/dm_et) | 1437 |

| | | |
|---|---|---|
| ProQuest (Health and medical collections) | (TI,AB(youth*) OR TI,AB(adolescen*) OR TI,AB("high school") OR TI,AB(junior*) OR TI,AB(young) OR TI,AB(student*) OR TI,AB(child) OR TI,AB(teenage*) OR MESH.EXACT(Adolescent)) AND (TI,AB(athlete*) OR TI,AB(player*) OR MESH.EXACT(Athletes)) AND (TI,AB(Rugby) OR TI,AB(Football) OR TI,AB("Collision sport*") OR TI,AB("Contact sport*") OR TI,AB("invasion sport*") OR TI,AB(tackle*) OR TI,AB(Soccer) OR TI,AB(Hockey) OR TI,AB(Lacrosse) OR TI,AB(Camogie) OR TI,AB(futsal) OR TI,AB(basketball) OR TI,AB(handball) OR TI,AB("ultimate frisbee") OR TI,AB("water polo") OR MESH.EXACT("Rugby") OR MESH.EXACT("Football") OR MESH.EXACT("Hockey") OR MESH.EXACT("Basketball") OR MESH.EXACT("Soccer")) AND (TI,AB(risk*) OR TI,AB(determinant*) OR TI,AB(factor*) OR TI,AB(influence*) OR TI,AB(predisposition*) OR TI,AB(predictor*) OR TI,AB(relationship*) OR TI,AB(causa*) OR TI,AB(association*) OR TI,AB(mechanism*) OR TI,AB(etiology) OR TI,AB(aetiology) OR TI,AB("odds ratio") OR TI,AB("hazard ratio") OR TI,AB("rate ratio") OR TI,AB(incidence) OR TI,AB(probability) OR TI,AB(epidemiology) OR TI,AB(prevalence) OR TI,AB(rate) OR MESH.EXACT(Risk) OR MESH.EXACT("Epidemiology")) AND (TI,AB(src) OR TI,AB(concussion*) OR TI,AB("brain injur*") OR TI,AB("head injur*") OR TI,AB(mtbi) OR MESH.EXACT("brain concussion")) | 768 |
| ProQuest (Theses and dissertations collections) | (TI,AB(youth*) OR TI,AB(adolescen*) OR TI,AB("high school") OR TI,AB(junior*) OR TI,AB(young) OR TI,AB(student*) OR TI,AB(child) OR TI,AB(teenage*) OR MESH.EXACT(Adolescent)) AND (TI,AB(athlete*) OR TI,AB(player*) OR MESH.EXACT(Athletes)) AND (TI,AB(Rugby) OR TI,AB(Football) OR TI,AB("Collision sport*") OR TI,AB("Contact sport*") OR TI,AB("invasion sport*") OR TI,AB(tackle*) OR TI,AB(Soccer) OR TI,AB(Hockey) OR TI,AB(Lacrosse) OR TI,AB(Camogie) OR TI,AB(futsal) OR TI,AB(basketball) OR TI,AB(handball) OR TI,AB("ultimate frisbee") OR TI,AB("water polo") OR MESH.EXACT("Rugby") OR | 140 |

| | | |
|---|---|---|
| | MESH.EXACT("Football") OR MESH.EXACT("Hockey") OR MESH.EXACT("Basketball") OR MESH.EXACT("Soccer")) AND (TI,AB(risk*) OR TI,AB(determinant*) OR TI,AB(factor*) OR TI,AB(influence*) OR TI,AB(predisposition*) OR TI,AB(predictor*) OR TI,AB(relationship*) OR TI,AB(causa*) OR TI,AB(association*) OR TI,AB(mechanism*) OR TI,AB(etiology) OR TI,AB(aetiology) OR TI,AB("odds ratio") OR TI,AB("hazard ratio") OR TI,AB("rate ratio") OR TI,AB(incidence) OR TI,AB(probability) OR TI,AB(epidemiology) OR TI,AB(prevalence) OR TI,AB(rate) OR MESH.EXACT(Risk) OR MESH.EXACT("Epidemiology")) AND (TI,AB(src) OR TI,AB(concussion*) OR TI,AB("brain injur*") OR TI,AB("head injur*") OR TI,AB(mtbi) OR MESH.EXACT("brain concussion”)) | |
