# Supplementary material for: Incidence and Risk Factors for Sport-Related Concussion in Female Youth Athletes Participating in Contact and Collision Invasion Sports: A Systematic Review
Source: Sports Med. 2024 Dec 8;55(2):393–418. doi: 10.1007/s40279-024-02133-x (PMC11947075; doi:10.1007/s40279-024-02133-x)
Supplement: Supplementary file 2 — Supplementary file2 (PDF 209 KB) [file 40279_2024_2133_MOESM2_ESM.pdf]

Incidence and Risk Factors for Sport-Related Concussion in Female Youth Athletes Participating in Contact and Collision Invasion Sports: A Systematic Review

Sports Medicine

Laura Ernst[1], Jessica Farley[1], and Nikki Milne[1]

[1] Faculty of Health Science and Medicine, Bond University, Qld, Australia 4226

* Corresponding Author: Laura Ernst, Email: laura.ernst@student.bond.edu.au

Online Resource 2. Study characteristics for all studies included in the systematic review

Author and Year	Study Design	Population (only as per review inclusion criteria)	Contact/collision sport/s	Competition year/s	Context	Utilised SRC definition or type of injury definition	SRC screening and assessment tools	Method of SRC diagnosis or reporting	Quality score	Quality rating
Barden et al. [76]	Descriptive epidemiology study	Single English high school, UK	Soccer and rugby union[a]	2015-2019	Match	24-hour time loss injury	-	Medical staff	67%	Good
Baron et al. [82]	Prospective cohort	NY public high schools and nationwide high school data, USA	Lacrosse	2009-2016 control 2017-2018 HG cohort	Match, practice and overall	Medical attention and time-loss injuries	-	Medical professional	67%	Good
Beaudouin et al. [75]	Cross-sectional observational design	480 European youth soccer teams from 8 European countries	Soccer	2018-2018	Match and practice combined	-	-	-	56%	Fair
Bretzin et al. [84]	Descriptive epidemiology study	755 Michigan high schools, USA	Basketball, soccer and lacrosse	2015-2016	Match and practice combined	Time-loss injuries	-	Medical doctor, doctor of osteopathic medicine, nurse practitioner or physician's assistant	78%	Good
Bretzin et al. [83]	Prospective longitudinal cohort study	All high schools in the MHSSA, USA	Soccer	2016-2019	Match and practice combined	Time-loss injuries	-	Physician, osteopath, nurse practitioner or physician's assistant	78%	Good
Cairo et al. [35]	Secondary analysis of a cross-sectional study (retrospective)	Students from 33 high schools in Alberta, Canada	Ringette, rugby union[a], field hockey, soccer, basketball, ice hockey[a] and lacrosse	2018-2019	Match and practice combined	SRC definition as outlined by Black et al. [129] in the Canadian S&R online survey	-	Self-reported	22%	Poor

Castile et al. [115]	Descriptive epidemiology study	Data from 100 nationally representative high schools, USA	Soccer and basketball	2005-2010	Match, practice and overall	Medical attention and time-loss injuries	-	AT or physician	78%	Good
Caswell et al. [74]	Descriptive epidemiology study	25 varsity and 25 junior varsity teams from 25 public high schools, USA	Lacrosse	2008-2009	Match	Medical attention injuries	-	AT	56%	Fair
Chun et al. [27]	Descriptive epidemiology study	92,966 athletes from 63 high schools across the state of Hawaii, USA	Soccer and basketball	2011-2017	Match and practice combined	SRC was defined to be consistent with diagnostic criteria as outlined by CISG [130]	ImPACT	AT	78%	Good
Clifton et al. [85]	Descriptive epidemiology study	Data from 100 nationally representative high schools, USA	Basketball	2005-2014	Match and practice	Medical attention and time-loss injuries	-	AT, physician, or other health care professional	67%	Good
Collins et al. [127]	Descriptive epidemiology study	121 boys' and girls' high school rugby clubs, USA	Rugby union[a]	2005-2006	Match and practice	Medical attention and time-loss injuries	-	Club physician, AT, personal physician, emergency department or urgent care facility	11%	Poor
Collins et al. [73]	Prospective cohort study	51 high schools across the USA	Soccer, basketball and lacrosse	2010-2011	-	-	-	AT	56%	Fair
Comstock et al. [86]	Retrospective cohort study	Data from 100 nationally representative high schools, USA	Soccer	2005-2014	Match and practice	Medical attention and time-loss injuries	-	AT or physician	78%	Good
Comstock et al. [87]	Retrospective cohort study	Data from 100 nationally representative high schools, USA	Lacrosse	2008-2019	Match and practice	Medical attention injuries	-	AT	67%	Good
Covassin et al. [88]	Descriptive epidemiology study	Student-athletes in grades 9 through 12 in Michigan, USA	Lacrosse, basketball, and soccer	2015-2016	Match and practice	Time-loss injuries	-	Medical professional	89%	Good

DiStefano et al. [89]	Descriptive epidemiology study	Data from 100 nationally representative high schools, USA	Soccer	2005-2014	Match and practice	Medical attention injuries	-	AT or physician	67%	Good
Eliason et al. [80]	Prospective cohort study	Players from youth ice hockey leagues in British Columbia and Alberta, Canada	Ice hockey[a]	2013-2018	Match and practice combined	SRC was defined to be in line with consensus statements put forward by McCrory et al. [131] and McCrory et al. [130]	SCAT-3, SCAT-5 or other SRC screening and/or assessment tool not defined	Sport medicine physician	89%	Good
Eliason et al. [77]	Prospective cohort study	Players from youth ice hockey leagues in British Columbia and Alberta, Canada	Ice hockey[a]	2013-2018	Match and practice combined	SRC was defined to be in line with consensus statements put forward by McCrory et al. [131] and McCrory et al. [130]	SCAT-3, SCAT-5 or other SRC screening and/or assessment tool not defined	Sport medicine physician	89%	Good
Gessel et al. [90]	Descriptive epidemiology study	Data from 100 nationally representative high schools, USA	Soccer and basketball	2005-2006	Match, practice and overall	Medical attention and time-loss injuries	-	AT or physician	67%	Good
Goldenberg and Hossler [123]	Prospective observational study	63 high schools representing 15 states across the USA	Lacrosse	-	Match and practice combined	Medical attention and time-loss injuries	-	Coach or AT	44%	Poor
Gomez et al. [122]	Prospective observational study	100 randomly selected Class 4A and 5A Texas public high schools, USA	Basketball	1993-1994	Match and practice combined	Medical attention and time-loss injuries	-	AT or physician	44%	Poor
Haarbauer-Krupa et al. [91]	Descriptive epidemiology study	Data from 100 nationally representative high schools and a large national convenience sample of over	Soccer, basketball, lacrosse, and field hockey	2009-2013	Match, practice and overall	Medical attention and time-loss injuries	-	AT, neurologist, healthcare professional, general physician, orthopaedic physician	67%	Good

		200 high schools, USA								
Herman et al. [92]	Descriptive epidemiology study (quasi-experimental comparison)	Female athletes from HG mandated schools across Florida, and non-HG mandated high school states across the USA	Lacrosse	2019-2021	Match, practice and overall	Medical attention injuries	-	AT, physician, or other healthcare professional	67%	Good
Hinton et al. [93]	Descriptive epidemiology study	23 high schools in the Fairfax County public school system, Virginia, and elite 300 girls' and 205 boys' summer lacrosse camps in Baltimore, Maryland, USA	Lacrosse	1999-2001	Match and practice combined	Medical attention and time-loss injuries	-	AT	67%	Good
Jingzhen et al. [94]	Descriptive epidemiology study	Data from 100 nationally representative high schools, USA	Soccer and basketball	2005-2016	Match and practice combined	Medical attention and time-loss injuries	-	AT or physician	67%	Good
Kerr et al. [96]	Descriptive epidemiology study	Data from 100 nationally representative high schools, USA	Soccer, basketball, lacrosse, and field hockey	2013-2018	Match, practice and overall	Medical attention injuries	-	AT or physician	67%	Good
Kerr et al. [95]	Descriptive epidemiology study	A convenience sample of nationally representative high schools, USA	Soccer	2012-2016	Match, practice and overall	Medical attention injuries	-	AT	67%	Good
Khodaee et al. [97]	Descriptive epidemiology study	Data from 100 nationally representative high schools, USA	Soccer	2005-2014	Match, practice and overall	Medical attention and time-loss injuries	-	AT or physician	78%	Good

Kriz et al. [72]	Prospective cohort study	180 high schools across 14 states in the USA	Field hockey	2009-2011	Match and practice combined	Medical attention and time-loss injuries	-	AT or physician	56%	Fair
Kriz et al. [71]	Retrospective analysis of a longitudinal prospective cohort study	206 high schools across 16 different states in the USA	Field hockey	2009-2013	Match and practice combined	Medical attention and time-loss injuries	-	AT or physician	56%	Fair
Kroshus et al. [98]	Cross-sectional survey	A random sample of coaches from Washington state public high schools, USA	Soccer	2012-2013	Match	-	-	Coach	78%	Good
Le Gall et al. [107]	Cohort study	French female elite soccer players at the Clairefontaine CNFE	Soccer	1998-2006	Match, practice and overall	All complaints and time-loss injuries	-	Physician	78%	Good
Lincoln et al. [126]	Descriptive epidemiology study	23 high schools in Fairfax County public school system, Virginia, USA	Lacrosse	2000-2003	Match and practice	Medical attention and time-loss injuries	-	AT	44%	Poor
Lincoln et al. [99]	Descriptive epidemiology study	25 high schools in a large public school system in Iowa, USA	Soccer, basketball, field hockey, and lacrosse	1997-2008	Match and practice combined	Medical attention injuries	Standardized Assessment of Concussion (CSMi Medical Solutions, Stoughton, California) and ImPACT	AT or physician	67%	Good
Lopez et al. [70]	Descriptive epidemiology study using a prospective observational cohort design	24 rugby 7s tournaments played within the USA	Rugby 7s[a]	2010-2014	Match	Medical attention and time-loss injuries	-	A study-trained healthcare provider, AT student, or AT	56%	Fair
Lynall et al. [116]	Descriptive epidemiology study	An average of 61 high schools sponsoring girls’	Field hockey	2008-2014	Match and practice	Medical attention for SRC	-	AT or physician	67%	Good

		field hockey, USA								
Marar et al. [29]	Descriptive epidemiology study	Data from 100 nationally representative high schools, USA	Soccer, basketball, field hockey, and lacrosse	2008-2010	Match, practice and overall	Medical attention injuries	-	AT or physician	78%	Good
Marshall et al. [100]	Descriptive epidemiology study using a retrospective analysis of an RCT	210 high schools in the eastern states (Pennsylvania, Virginia, District of Columbia, North Carolina, and South Carolina), USA	Soccer, lacrosse, and ice hockey[a]	1999-2001	Match and practice combined	SRC definition utilised a combination of symptomology and self-reporting scales outlined in Kelly and Rosenberg [132], Piland et al. [133] and Piland et al. [134]	-	AT	78%	Good
McGuine et al. [103]	Cluster randomised control trial	88 high schools in the USA	Soccer	2016-2018	Match and practice combined	SRC definition as provided by the National Athletic Trainers' Association Position Statement [135]	SCAT-3	AT	78%	Good
Messina et al. [125]	Descriptive epidemiology study	100 class 4A and 5A public high school in Texas, USA	Basketball	1993-1994 (females) and 1006-1997 (males)	Match and practice combined	Medical attention and time-loss injuries	-	AT or physician	44%	Poor
Murata et al. [101]	Descriptive epidemiology study	67 Hawaiʻi high school athletic programs, USA	Basketball and soccer	2010-2016	Match and practice combined	-	ImPACT	AT and neuropsychologist	78%	Good
O'Connor et al. [102]	Descriptive epidemiology study	147 high schools from 26 states in the USA	Field hockey, basketball, lacrosse, and soccer	2001-2014	Match, practice and overall	SRC definition as outlined by each athletic trainers local institution or state guideline. Where no guidelines existed the SRC definition from McCrory et al. [131] was provided	-	Healthcare provider	67%	Good

O'Kane et al. [104]	Prospective cohort study	33 elite (select and premier) teams from 4 youth soccer clubs in the Puget Sound region of Washington State, USA	Soccer	2008-2012	Match and practice combined	SRC was defined to be consistent with diagnostic criteria as outlined by the CISG [136]	-	Physician, nurse practitioner, physician assistant, AT or self-reported	67%	Good
O'Kane et al. [65]	Prospective cohort study	33 elite (select and premier) teams from 4 youth soccer clubs in the Puget Sound region of Washington State, USA	Soccer	2008-2012	Match and practice combined	SRC was defined to be consistent with diagnostic criteria as outlined by the CISG [136]	-	Physician, nurse practitioner, physician assistant, AT or self-reported	56%	Fair
Owoeye et al. [105]	Prospective cohort study	Athletes from 52 high schools and 23 basketball clubs in Calgary, Alberta, Canada	Basketball	2006-2017	Match and practice combined	All complaints injuries	-	AT or medical practitioner	67%	Good
Pasanen et al. [124]	Prospective cohort study	Seven adolescent floorball teams from three sports clubs from Tampere City district, Finland	Floorball	2011-2014	Match and practice combined	Time-loss injuries	-	Self-reported	44%	Poor
Pierpoint et al. [69]	Descriptive epidemiology study	A national sample of 100 schools with ATs and a sample of Chicago public high schools without ATs, USA	Soccer and basketball	2006-2009	Match and practice combined	Medical attention and time-loss injuries	-	AT, physician or coach	56%	Fair
Pierpoint et al. [106]	Descriptive epidemiology study	An average of 55 high schools sponsoring girls' lacrosse across the USA	Lacrosse	2008-2014	Match, practice and overall	Medical attention injuries	-	AT or physician	67%	Good

Powell and Barber-Foss [117]	Observational cohort study	Varsity athletes from 235 high schools across the USA.	Basketball, field hockey, and soccer	1995-1997	Match, practice and overall	Medical attention injuries	-	AT	78%	Good
Rauh et al. [108]	Observational study	Data from 235 selected schools among the 50 states in the USA	Basketball, field hockey, and soccer	1995-1997	Match and practice combined	Time-loss injuries	-	AT	67%	Good
Rechel et al. [109]	Prospective injury surveillance study	Data from 100 nationally representative high schools, USA	Soccer and basketball	2005-2006	Match, practice and overall	Medical attention and time-loss injuries	-	AT or physician	78%	Good
Reeschske et al. [78]	Prospective longitudinal study	Four youth soccer teams active in the three highest German junior leagues (Westfalen-,Regional-, Bundesliga)	Soccer	2019-2021	Match, practice and overall	-	-	-	67%	Good
Rivara et al. [67]	Cohort study	20 high school from Washington State, USA	Soccer	2012	Match and practice	SRC was defined to be in line with previous studies and consensus statements put forward by McCrory et al. [136] and McCrory et al. [131]	SCAT-2	Self-reported	56%	Fair
Rosenthal et al. [118]	Descriptive epidemiology study	Data from 100 nationally representative high schools, USA	Soccer and basketball	2005-2012	Match and practice combined	Medical attention and time-loss injuries	-	AT or physician	78%	Good
Schallmo et al. [110]	Descriptive epidemiology study	Data from 100 nationally representative high schools, USA	Soccer and basketball	2005-2014	Match and practice combined	Medical attention and time-loss injuries	-	AT or physician	78%	Good
Schneider et al. [111]	Prospective cohort study	44 elite (top 20% by division of	Ice hockey[a]	2011-2012	Match and	SRC was defined to be consistent with	SCAT-2, ImPACT, and	Medical practitioner	67%	Good

		play) youth ice hockey teams from Calgary and Edmonton, Canada			practice combined	diagnostic criteria as outlined by the CISG [136]	BASC-2			
Shill et al. [66]	Prospective cohort study	High schools from the Calgary Senior High School Athletics Association, USA	Rugby union[a]	2018-2019	Match and practice	Medical attention and time-loss injuries	-	Physician or unclear	56%	Fair
Shill et al. [79]	Prospective cohort study	High schools from the Calgary Senior High School Athletics Association, USA	Rugby union[a]	2018-2019	Match and practice	SRC was defined to be in line with consensus statements put forward by McCrory et al. [130]	-	Sport medicine physician	67%	Good
Smith et al. [68]	Prospective comparative analysis	Hockey tournaments in Minnesota, USA	Ice hockey[a]	2013-2014	Match	"A concussion was defined as a brain injury or transient neurological dysfunction resulting from a biomechanical force" Smith et al. [68]	SCAT-3, the PCSS, Delayed Recall, and the Maddox Scale	AT or emergency medical technician	56%	Fair
Tuominen et al. [112]	Descriptive epidemiology study	World ice hockey championships consisting of 19 different countries	Ice hockey[a]	2006-2014	Match	Medical attention injuries	-	Physician	67%	Good
Tuominen et al. [119]	Prospective observational design	World Championship tournaments and Olympic Games over 9 ice hockey seasons	Ice hockey[a]	2006-2015	Match	Medical attention and time-loss injuries	SCAT	Physician	78%	Good
Warner et al. [113]	Descriptive epidemiology study	Data from 100 nationally representative	Lacrosse	2008-2016	Match, practice and overall	Medical attention injuries	-	AT or physician	67%	Good

		high schools, USA							
Williamson et al. [81]	Cross-sectional study	Teams competing in Under 15 AA elite club games in the City of Calgary (Alberta, Canada)	Ice hockey[a]	2020-2022	Match	"Suspected injuries were identified if (i) gameplay was stopped for suspected injury, (ii) a player remained on the ice surface for >15s, or (iii) a player received medical attention. SRC was suspected if the above criteria was met and at least 1 of 17 validated criteria of visual SRC signs were reached (eg, dazed, lying motionless, clutching head)." Williamson et al. [81]	"Video analysis of suspected SRC based on validated SRC criteria previously put forward by West et al. (2022) and Davis et al. (2019)" Williamson et al. [81]	Trained video-analysers with experience coaching and/or playing ice hockey were responsible for identifying suspected SRC	67%	Good
Xiang et al. [114]	Descriptive epidemiology study	Data from 100 nationally representative high schools, USA	Lacrosse	2008-2012	Match, practice and overall	Medical attention for SRC	-	AT or physician	67%	Good
Yard et al. [121]	Descriptive epidemiology study	Data from 100 nationally representative high schools, USA	Soccer	2005-2007	Match and practice combined	Medical attention and time-loss injuries	-	AT or physician	78%	Good
Yard and Comstock [120]	Prospective cohort study	Data from 100 nationally representative high schools, USA	Soccer and basketball	2005-2008	Match and practice combined	Medical attention and time-loss injuries	American Academy of Neurology and Prague guidelines for concussion grading and return-to-play	AT or physician	78%	Good

- not reported or not investigated, [a] collision sports, AT athletic trainer, BASC-2 Behavior Assessment System for Children Second Edition, CISG Concussion in Sport Group, CSMi computer sports medicine incorporated, HG headgear, ImPACT Immediate Post-Concussion Assessment and Cognitive Testing, MHSSA Michigan High School Athletic Association, NY New York, PCSS Post-Concussion Symptom Scale, S&R sport and recreation, s seconds, SCAT Sport Concussion Assessment Tool, SCAT-2 Sport Concussion Assessment Tool-2, SCAT-3 Sport Concussion Assessment Tool-3, SCAT-5 Sport Concussion Assessment Tool-5, SRC sport-related concussion, USA United States of America
