# Supplementary material for: Incidence and Risk Factors for Sport-Related Concussion in Female Youth Athletes Participating in Contact and Collision Invasion Sports: A Systematic Review
Source: Sports Med. 2024 Dec 8;55(2):393–418. doi: 10.1007/s40279-024-02133-x (PMC11947075; doi:10.1007/s40279-024-02133-x)
Supplement: Supplementary file 3 — Supplementary file3 (PDF 55 KB) [file 40279_2024_2133_MOESM3_ESM.pdf]

Incidence and Risk Factors for Sport-Related Concussion in Female Youth Athletes Participating in Contact and Collision Invasion Sports: A Systematic Review

Sports Medicine

Laura Ernst[1], Jessica Farley[1], and Nikki Milne[1]

[1] Faculty of Health Science and Medicine, Bond University, Qld, Australia 4226

* Corresponding Author: Laura Ernst, Email: laura.ernst@student.bond.edu.au

Online Resource 3. Number of sport-related concussions and incidence rates sustained by youth female athletes during practice/training in contact/collision invasion sports

Study	Sample size	Number of SRCs	Method of exposure	IR	95% CI
SOCCER					
**Castile et al. [115]**	-	31[b]	/100000 AE	5.40	-
	-	4[c]		0.70	-
**Covassin et al. [88]**	-	57	/100 player seasons	0.43[a]	-
**DiStefano et al. [89]**	-	90	/1000 AE	0.09	0.07-0.11
**Gessel et al. [90]**	-	-	/1000 AE	0.09	-
**Haarbauer-Krupa et al. [91]**	-	74	/10000 AE	1.20	0.95-1.52[a]
**Kerr et al. [96]**	-	191	/10000 AE	2.14	1.85-2.47[a]
**Kerr et al. [95]**	-	168	/10000 AE	4.02	3.41-4.63
**Le Gall et al. [107]**	n = 119	1	/1000 hours	0.01	-
**Marar et al. [29]**	-	26	/10000 AE	0.80	-
**O'Connor et al. [102]**	-	40	/10000 AE	2.96	2.04-3.88
**Powell and Barber-Foss [117]**	-	-	/1000 AE	0.05	0.02-0.08
**Rechel et al. [109]**	-	10[a]	/1000 AE	0.11[a]	-
**Rivara et al. [67]**	n = 288	5	/1000 AE	0.80	0.30-1.90
BASKETBALL					
**Castile et al. [115]**	-	33[b]	/100000 AE	5.10	-
	-	9[c]		1.40	-
**Clifton et al. [85]**	-	122	/1000 AE	0.11	0.09-0.13
**Covassin et al. [88]**	-	114	/100 player seasons	0.73[a]	-
**Gessel et al. [90]**	-	-	/1000 AE	0.06	-
**Haarbauer-Krupa et al. [91]**	-	103	/10000 AE	1.40	1.17-1.74[a]
**Kerr et al. [96]**	-	142	/10000 AE	1.55	1.31-1.83[a]
**Marar et al. [29]**	-	22	/10000 AE	0.60	-
**O'Connor et al. [102]**	-	47	/10000 AE	2.22	1.59-2.86
**Powell and Barber-Foss [117]**	-	20	/1000 AE	0.07	0.04-0.10
**Rechel et al. [109]**	-	6[a]	/1000 AE	0.05[a]	0.02-0.10[a]

**LACROSSE**					
**Baron et al. [82]**	n = 1585	1[d]	/1000 AE	0.07	0.00-0.38[a]
	-	74		0.16	-
**Comstock et al. [87]**	-	106	/10000 AE	1.56	1.28-1.89[a]
**Covassin et al. [88]**	-	6	/100 player seasons	0.22[a]	-
**Haarbauer-Krupa et al. [91]**	-	29	/10000 AE	1.40	0.96-2.05
**Herman et al. [92]**	-	52	/1000 AE	0.20[a]	0.15-0.26[a]
**Kerr et al. [96]**	-	57	/10000 AE	1.66	-
**Marar et al. [29]**	-	15	/10000 AE	1.30	-
**O'Connor et al. [102]**	-	26	/10000 AE	3.44	2.12-4.76
**Pierpoint et al. [106]**	-	52	/1000 AE	0.16	0.11-0.20
**Warner et al. [113]**	-	79	/10000 AE	1.70	-
**Xiang et al. [114]**	-	33[a]	/1000 AE	0.16	-
**RUGBY UNION[j]**					
**Shill et al. [66]**	n = 361	16	/1000 practice hours	1.00	0.70-1.40
**Shill et al. [79]**	n = 361	11[g]	/1000 practice hours	0.7	0.4-1.2
		7[h]		0.4	0.2-0.8
		4[i]		0.3	0.1-0.5
**FIELD HOCKEY**					
**Haarbauer-Krupa et al. [91]**	-	32	/10000 AE	1.30	0.86-1.78
**Kerr et al. [96]**	-	12	/10000 AE	0.87	0.54-1.32[a]
**Lynall et al. [116]**	-	45	/1000 AE	0.12	0.08-0.15
**Marar et al. [29]**	-	22	/10000 AE	1.40	-
**O'Connor et al. [102]**	-	27	/10000 AE	2.47	1.54-3.40
**Powell and Barber-Foss [117]**	-	-	/1000 AE	0.02	0.00-0.05
**ICE HOCKEY[j]**					
**Eliason et al. [77]**	-	-[e]	/1000 practice hours	0.40	0.19-0.84
	-	-[f]		0.30	0.10-0.93

- not reported or not investigated, **[a]** Calculated using raw data extracted, [b] Sports-related concussion first-time occurrence (i.e., new), [c] Recurrent sports-related concussion**,** [d] Headgear cohort, [e] Under 15 cohort, [f] Under 18 cohort, [g] tackle related SRCs, [h] ball-carrier tackle related SRCs, [i] tackler tackle related SRCs, [j] collision sports, AE athletic exposure, CI confidence interval, IR incidence rate, SRC sport-related concussion
