# Supplementary material for: Incidence and Risk Factors for Sport-Related Concussion in Female Youth Athletes Participating in Contact and Collision Invasion Sports: A Systematic Review
Source: Sports Med. 2024 Dec 8;55(2):393–418. doi: 10.1007/s40279-024-02133-x (PMC11947075; doi:10.1007/s40279-024-02133-x)
Supplement: Supplementary file 4 — Supplementary file4 (PDF 61 KB) [file 40279_2024_2133_MOESM4_ESM.pdf]

Incidence and Risk Factors for Sport-Related Concussion in Female Youth Athletes Participating in Contact and Collision Invasion Sports: A Systematic Review

Sports Medicine

Laura Ernst[1], Jessica Farley[1], and Nikki Milne[1]

[1] Faculty of Health Science and Medicine, Bond University, Qld, Australia 4226

A Corresponding Author: Laura Ernst, Email: laura.ernst@student.bond.edu.au

Online Resource 4. Number of sport-related concussions and incidence rates sustained by youth female athletes during matches in contact/collision invasion sports

Study	Sample size	No. of SRCs	Method of exposure	IR	95% CI
SOCCER					
**Barden et al. [76]**	-	4	/1000 match hours	4.00	2.00-11.00
**Beaudouin et al. [75]**	-	1	/1000 hours	1.11[a]	0.03-6.18[a]
**Castile et al. [115]**	-	178[b]	/100000 AE	73.50	-
	-	29[c]		12.00	-
**Covassin et al. [88]**	-	349	/100 player seasons	2.61[a]	-
**DiStefano et al. [89]**	-	537	/1000 AE	1.28	1.18-1.39
**Gessel et al. [90]**	-	-	/1000 AE	0.97	-
**Haarbauer-Krupa et al. [91]**	-	439	/10000 AE	16.20	14.76-17.84[a]
**Kerr et al. [96]**	-	864	/10000 AE	21.80	20.37-23.30[a]
**Kerr et al. [95]**	-	743	/10000 AE	40.03	37.52-43.34
**Le Gall et al. [107]**	n = 119	1	/1000 hours	0.10	0.00-0.57[a]
**Marar et al. [29]**	-	133	/10000 AE	9.20	-
**O'Connor et al. [102]**	-	66	/10000 AE	17.16	13.02-21.30
**Powell and Barber-Foss [117]**	-	-	/1000 AE	0.71	0.53-0.88
**Rechel et al. [109]**	-	42[a]	/1000 AE	0.98[a]	-
**Reeschske et al. [78]**	n = 39	1	/1000 match hours	1.27[a]	0.03-7.06[a]
**Rivara et al. [67]**	n = 288	27	/1000 AE	10.00	6.60-14.50
BASKETBALL					
**Castile et al. [115]**	-	120[b]	/100000 AE	44.10	-
	-	18[c]		6.60	-
**Clifton et al. [85]**	-	357	/1000 AE	0.74	0.66-0.82
**Covassin et al. [88]**	-	340	/100 player seasons	2.19[a]	-
**Gessel et al. [90]**	-	-	/1000 AE	0.60	-
**Haarbauer-Krupa et al. [91]**	-	287	/10000 AE	9.00	8.02-10.14[a]
**Kerr et al. [96]**	-	504	/10000 AE	12.10	11.07-13.21[a]
**Marar et al. [29]**	-	85	/10000 AE	5.50	-
**O'Connor et al. [102]**	-	81	/10000 AE	10.52	8.23-12.82

**Rechel et al. [109]**	-	36[a]	/1000 AE	0.68[a]	0.47-0.94[a]
**RUGBY UNION[j]**					
**Barden et al. [76]**	-	15	/1000 match hours	27.00	16.00-44.00
**Shill et al. [66]**	n = 361	62	/1000 match hours	37.50	26.80-52.30
**Shill et al. [79]**	n = 361	49[g]	/1000 match hours	29.6	20.3-43.1
		19[h]		11.5	7.0-19.0
		30[i]		18.1	11.5-28.6
**LACROSSE**					
**Baron et al. [82]**	n = 1585	1[d]	/1000 AE	0.13	0.00-0.73[a]
	-	182		0.86	-
**Caswell et al. [74]**	n = approximately 2500	21	/1000 games	39.70[a]	-
**Comstock et al. [87]**	-	278	/10000 AE	9.16	8.12-10.30[a]
**Covassin et al. [88]**	-	24	/100 player seasons	0.87[a]	-
**Haarbauer-Krupa et al. [91]**	-	78	/10000 AE	8.40	6.61-10.43[a]
**Herman et al. [92]**	-	89	/1000 AE	0.90[a]	0.72-1.11[a]
**Kerr et al. [96]**	-	152	/10000 AE	10.01	-
**Marar et al. [29]**	-	45	/10000 AE	8.60	-
**O'Connor et al. [102]**	-	30	/10000 AE	11.75	7.55-15.95
**Pierpoint et al. [106]**	-	125	/1000 AE	0.83	0.69-0.98
**Warner et al. [113]**	-	194	/10000 AE	9.20	-
**Xiang et al. [114]**	-	72[a]	/1000 AE	0.78	-
**RUGBY 7s[j]**					
**Lopez et al. [70]**	n = 732	7[a]	/1000 hours	15.42	6.20-31.77[a]
**FIELD HOCKEY**					
**Haarbauer-Krupa et al. [91]**	-	72	/10000 AE	6.10	4.77-7.67[a]
**Kerr et al. [96]**	-	77	/10000 AE	6.51	5.14-8.13[a]
**Lynall et al. [116]**	-	107	/1000 AE	0.59	0.48-0.71
**Marar et al. [29]**	-	29	/10000 AE	4.10	-
**O'Connor et al. [102]**	-	39	/10000 AE	9.83	6.74-12.91
**Powell and Barber-Foss [117]**	-	-	/1000 AE	0.29	0.12-0.46
**ICE HOCKEY[j]**					
**Eliason et al. [80]**	-	-[e]	/1000 match hours	2.62	1.93-3.56
		-[f]		2.34	1.51-3.63
**Smith et al. [68]**	n = 328	1	/1000 match hours	3.10[a]	0.08-17.25[a]

**Tuominen et al. [112]**	n = 1968	-	/1000 player games	1.40	-
**Tuominen et al. [119]**	-	15	/1000 match hours	4.70	2.63-7.75[a]
			/1000 player games	1.40	-
**Williamson et al. [81]**	-	3	/100 team minutes	0.25	0.09-0.73

- not reported or not investigated, [a] Calculated using raw data extracted, [b] Sport-related concussion first-time occurrence (i.e., new), [c] Recurrent sport-related concussion, [d] Headgear cohort, [e] Under 15 cohort, [f] Under 18 cohort, [g] tackle related SRCs, [h] ball-carrier tackle related SRCs, [i] tackler tackle related SRCs, [j] collision sports, AE athletic exposure, CI confidence interval, IR incidence rate, SRC sport-related concussion
