# Supplementary material for: Incidence and Risk Factors for Sport-Related Concussion in Female Youth Athletes Participating in Contact and Collision Invasion Sports: A Systematic Review
Source: Sports Med. 2024 Dec 8;55(2):393–418. doi: 10.1007/s40279-024-02133-x (PMC11947075; doi:10.1007/s40279-024-02133-x)
Supplement: Supplementary file 5 — Supplementary file5 (PDF 82 KB) [file 40279_2024_2133_MOESM5_ESM.pdf]

Incidence and Risk Factors for Sport-Related Concussion in Female Youth Athletes Participating in Contact and Collision Invasion Sports: A Systematic Review

Sports Medicine

Laura Ernst[1], Jessica Farley[1], and Nikki Milne[1]

[1] Faculty of Health Science and Medicine, Bond University, Qld, Australia 4226

* Corresponding Author: Laura Ernst, Email: laura.ernst@student.bond.edu.au

Online Resource 5. Number of sport-related concussions and incidence rates sustained by youth female athletes during practice/training and matches combined in contact/collision invasion sports

Study	Sample size	No. of SRCs	Method of Exposure	IR	95% CI
**SOCCER**					
**Bretzin et al. [84]**	n = 13367	316	/100 player seasons	2.40	2.10-2.60
**Bretzin et al. [83]**	n = 39637	950	/100 athletes per seasons	2.40[a]	-
**Castile et al. [115]**	-	209[b]	/100000 AE	25.80	-
	-	33[c]		4.10	-
**Chun et al. [27]**	n = 8080	579	/1000 AE	1.10	1.01-1.19
**Comstock et al. [86]**	-	627	/10000 AE	4.50	-
		157[r]		1.13[a]	-
**Covassin et al. [88]**	-	-	/100 player seasons	3.04[a]	2.74-3.33
**Gessel et al. [90]**	-	51	/1000 AE	0.36	-
**Haarbauer-Krupa et al. [91]**	-	513	/10000 AE	5.80	5.30-6.30[a]
**Jingzhen et al. [94]**	-	911	/100000 AE	54.90	52.00-58.00
**Kerr et al. [96]**	-	1055	/10000 AE	8.19	7.70-8.70[a]
**Kerr et al. [95]**	-	911	/10000 AE	15.14	14.15.16.12
**Khodaee et al. [97]**	-	616[a]	/1000 AE	0.44[a]	-
**Le Gall et al. [107]**	n = 119	2	/1000 hours	0.02	0.00-0.07[a]
**Lincoln et al. [99]**	-	195	/1000 AE	0.35	0.31-0.41[a]
**Marar et al. [29]**	-	159	/10000 AE	3.40	-
**Marshall et al. [100]**	n = 1619	31	/100000 AE	13.40	8.70-18.10
**Murata et al. [101]**	-	495[a]	/1000 AE	0.93[a]	0.85-1.01[a]
**O'Connor et al. [102]**	-	106	/10000 AE	6.11	4.94-7.27
**O'Kane et al. [65]**	-	19[s]	/1000 hours	1.20	0.70-1.90
	-	17[t]		1.60	0.90-2.50
**O'Kane et al. [104]**	-	36[a]	/1000 hours	1.36[a]	0.95-1.89[a]
**Pierpoint et al. [69]**	-	150	/1000 AE	0.26[a]	0.22-0.31[a]

**Powell and Barber-Foss [117]**	-	76	/1000 AE	0.23	0.18-0.28
**Rauh et al. [108]**	-	3[c]	/100 player seasons	0.05[a]	-
		68[b]		1.02[a]	-
**Rechel et al. [109]**	-	54[a]	/1000 AE	0.38[a]	-
**Reeschske et al. [78]**	n = 39	1	/1000 hours	0.33[a]	0.01-1.83[a]
**Rivara et al. [67]**	n = 288	32	/1000 AE	3.60	2.40-5.10
**Rosenthal et al. [118]**	-	134[j]	/1000 AE	0.28	0.24-0.33
	-	-[k]		0.73	0.60-0.89
**Schallmo et al. [110]**	-	-[l]	/10000 AE	3.58	-
		-[m]		9.10	-
**Yard et al. [121]**	-	91[a]	/1000 AE	0.30[a]	-
**Yard and Comstock [120]**	-	-	/100000 AE	28.00	-
**BASKETBALL**					
**Bretzin et al. [84]**	n = 15558	387	/100 player seasons	2.50	2.20-2.70
**Castile et al. [115]**	-	153[b]	/100000 AE	16.70	-
		27[c]		2.90	-
**Chun et al. [27]**	n = 5399	381	/1000 AE	1.09	0.98-1.19
**Clifton et al. [85]**	-	479	/1000 AE	0.30[a]	-
**Covassin et al. [88]**	-	-	/100 player seasons	2.92	2.65-3.19
**Gessel et al. [90]**	-	40	/1000 AE	0.21	-
**Gomez et al. [122]**	n = 890	8	/1000 hours	0.08[a]	0.03-0.15[a]
**Haarbauer-Krupa et al. [91]**	-	390	/10000 AE	3.80	3.41-4.17[a]
**Jingzhen et al. [94]**	-	653	/100000 AE	34.60	32.50-36.60
**Kerr et al. [96]**	-	646	/10000 AE	4.85	4.48-5.24[a]
**Lincoln et al. [99]**	-	120	/1000 AE	0.16	0.14-0.20[a]
**Marar et al. [29]**	-	107	/10000 AE	2.10	-
**Messina et al. [125]**	n = 890	8	/1000 hours	0.07	0.03-0.13[a]
**Murata et al. [101]**	-	328[a]	/1000 AE	0.91	0.81-1.01[a]
**O'Connor et al. [102]**	-	128	/10000 AE	4.44	3.67-5.20
**Owoeye et al. [105]**	n = 200	8	/1000 hours	0.64	0.27-1.25
**Pierpoint et al. [69]**	-	112	/1000 AE	0.18[a]	0.15-0.21[a]
**Powell and Barber-Foss [117]**	-	63	/1000 AE	0.16	0.12-0.21
**Rauh et al. [108]**	-	4[ac]	/100 player seasons	0.07[a]	-
		54[ab]		0.89[a]	-

**Rechel et al. [109]**	-	42[a]	/1000 AE	0.23[a]	0.16-0.31[a]
**Rosenthal et al. [118]**	-	112[j]	/1000 AE	0.20	0.17-0.24
		-[k]		0.37	0.28-0.47
**Schallmo et al. [110]**	-	-[l]	/10000 AE	2.39	-
		-[m]		4.23	-
**Yard and Comstock [120]**	-	-	/10000 AE	16.60	-
**RUGBY UNION[u]**					
**Cairo et al. [35]**	n = 59	13[a]	/100 participants per year	15.30	-
**Collins et al. [127]**	-	11[a]	/1000 AE	0.58[a]	-
**Shill et al. [66]**	n = 361	78[a]	/100 player seasons	18.50	-
			/1000 hours	4.04[a]	3.48-5.50[a]
**Shill et al. [79]**	n = 361	60[g]	/1000 hours	3.39[a]	2.59-4.36[a]
		26[h]		1.47[a]	0.96-2.15[a]
		34[i]		1.92[a]	1.33-2.68[a]
**LACROSSE**					
**Baron et al. [82]**	-	258	/1000 AE (opponents were AE games only)	0.37[a]	0.32-0.41[a]
**Bretzin et al. [84]**	n = 2775	23	/100 player seasons	0.80	0.50-1.20
**Comstock et al. [87]**	-	384	/10000 AE	3.91	3.52-4.32[a]
**Covassin et al. [88]**	-	-	/100 player seasons	1.08	0.69-1.47
**Goldenberg and Hossler [123]**	n = 7263	37[a]	/1000 AE	0.10[a]	0.07-0.14
**Haarbauer-Krupa et al. [91]**	-	107	/10000 AE	3.60	3.20-4.10[a]
**Herman et al. [92]**	-	141	/1000 AE	0.39	0.33-0.46
**Hinton et al. [93]**	-	14[n]	/1000 AE	0.10	0.05-0.16[a]
	n = 1141	1[o]		0.13	0.00-0.70[a]
**Kerr et al. [96]**	-	209	/10000 AE	4.22	-
**Lincoln et al. [126]**	n = 3566	45	/1000 AE	0.21	0.16-0.29[a]
**Lincoln et al. [99]**	-	114	/1000 AE	0.20	0.17-0.25[a]
**Marar et al. [29]**	-	60	/10000 AE	3.50	-
**Marshall et al. [100]**	n = 504	21	/100000 AE	21.00	12.00-30.00
**O'Connor et al. [102]**	-	56	/10000 AE	5.54	4.09-6.99
**Pierpoint et al. [106]**	-	177	/1000 AE	0.37[a]	0.32-0.43
**Warner et al. [113]**	-	273	/10000 AE	4.00	-

**Xiang et al. [114]**	-	105[a]	/1000 AE	0.35	-
**FIELD HOCKEY**					
**Cairo et al. [35]**	n = 20	4[a]	/100 participants per year	10.00	-
**Haarbauer-Krupa et al. [91]**	-	104	/10000 AE	2.80	2.28-3.39[a]
**Kerr et al. [96]**	-	99	/10000 AE	2.66	2.17-3.14[a]
**Kriz et al. [72]**	-	93	/1000 AE	0.28[a]	-
**Kriz et al. [71]**	-	209	/1000 AE	0.34	0.29-0.38[a]
**Lincoln et al. [99]**	-	58	/1000 AE	0.10	0.08-0.13[a]
**Lynall et al. [116]**	-	152[a]	/1000 AE	0.27	0.23-0.31[a]
**Marar et al. [29]**	-	51	/10000 AE	2.20	-
**O'Connor et al. []**	-	66	/10000 AE	4.42	3.36-5.49
**Powell and Barber-Foss [117]**	-	13	/1000 AE	0.09	0.04-0.15
**Rauh et al. [108]**	-	1[ac]	/100 player seasons	0.04[a]	-
		11[ab]		0.39[a]	-
**RINGETTE**					
**Cairo et al. [35]**	n = 21	7[a]	/100 participants per year	19.00	-
**ICE HOCKEY[u]**					
**Schneider et al. [111]**	n = 50	9[p]	/1000 hours	1.39	0.97-1.99
	n = 69	13[q]		1.16	0.90-1.50
**FLOORBALL**					
**Pasanen et al. [124]**	n = 75	3	/1000 hours	0.08	0.00-0.18
**SOCCER, BASKETBALL, & LACROSSE**					
**Collins et al. [73]**	-	11	/10000 AE	4.90	-

- not reported or not investigated, [a] Calculated using raw data extracted, [b] Sport-related concussion first-time occurrence (i.e., new), [c] Recurrent sport-related concussion, [d] Headgear cohort, [e] Under 15 cohort, [f] Under 18 cohort, [g] tackle related SRCs, [h] ball-carrier tackle related SRCs, [i] tackler tackle related SRCs, [j] cohort between 2005-2008, [k] cohort between 2011-2012, [l] cohort between 2005-2006, [m] cohort between 2014-2015, [n] cohort from high schools in Fairfax County Public school system Virginia United States of America, [o] cohort from summer lacrosse camps in Baltimore Maryland United States of America, [p] cohort of bantam aged female youth ice hockey players, cohort of midget aged female youth ice hockey players, [r] heading related SRC, [s] cohort of under 14 elite youth female soccer athletes from Puget Sound region in Washington State United States of America, [t] cohort of under 15 elite youth female soccer athletes from Puget Sound region in Washington State United States of America, [u] collision sports, AE athletic exposure, CI confidence interval, IR incidence rate, SRC sport-related concussion
