# Supplementary material for: Incidence and Risk Factors for Sport-Related Concussion in Female Youth Athletes Participating in Contact and Collision Invasion Sports: A Systematic Review
Source: Sports Med. 2024 Dec 8;55(2):393–418. doi: 10.1007/s40279-024-02133-x (PMC11947075; doi:10.1007/s40279-024-02133-x)
Supplement: Supplementary file 6 — Supplementary file6 (PDF 382 KB) [file 40279_2024_2133_MOESM6_ESM.pdf]

Incidence and Risk Factors for Sport-Related Concussion in Female Youth Athletes Participating in Contact and Collision Invasion Sports: A Systematic Review

Sports Medicine

Laura Ernst[1], Jessica Farley[1], and Nikki Milne[1]

[1] Faculty of Health Science and Medicine, Bond University, Qld, Australia 4226

* Corresponding Author: Laura Ernst, Email: laura.ernst@student.bond.edu.au

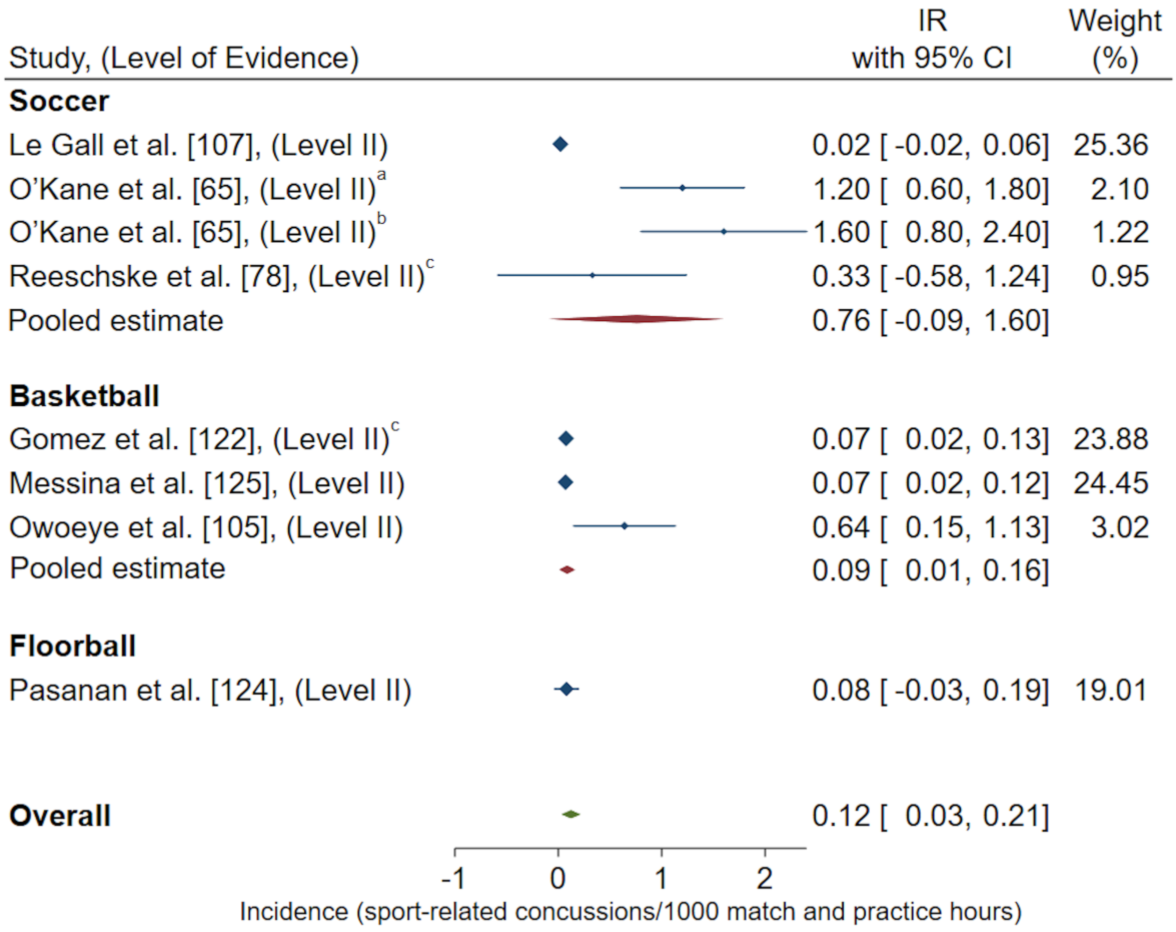

Online Resource 6. Pooled sport-related concussion incidence rate for contact invasion sports measured per 1000 match and practice hours combined. Presented in chronological order according to publication year. [a] Cohort = Under 14 elite female soccer athletes in the Puget Sound region of Washington State (United States of America), [b] cohort = Under 15 elite female soccer athletes in the Puget Sound region of Washington State (United States of America), [c] calculated using raw data extracted, CI confidence interval, IR incidence rate, Level II a prospective cohort study or a randomised controlled trial
