# Supplementary material for: Incidence and Risk Factors for Sport-Related Concussion in Female Youth Athletes Participating in Contact and Collision Invasion Sports: A Systematic Review
Source: Sports Med. 2024 Dec 8;55(2):393–418. doi: 10.1007/s40279-024-02133-x (PMC11947075; doi:10.1007/s40279-024-02133-x)
Supplement: Supplementary file 7 — Supplementary file7 (PDF 162 KB) [file 40279_2024_2133_MOESM7_ESM.pdf]

Incidence and Risk Factors for Sport-Related Concussion in Female Youth Athletes Participating in Contact and Collision Invasion Sports: A Systematic Review

Sports Medicine

Laura Ernst[1], Jessica Farley[1], and Nikki Milne[1]

[1] Faculty of Health Science and Medicine, Bond University, Qld, Australia 4226

* Corresponding Author: Laura Ernst, Email: laura.ernst@student.bond.edu.au

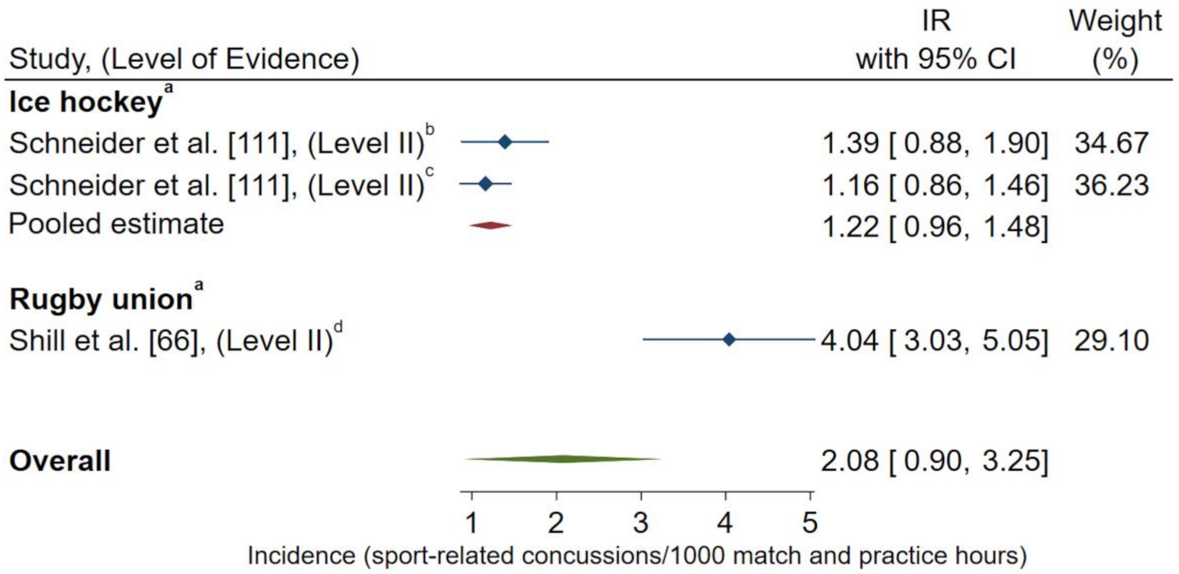

Online Resource 7. Pooled sport-related concussion incidence rate for collision invasion sports measured per 1000 match and practice hours combined. Presented in chronological order according to publication year. [a] Collision sports, [b] cohort = Bantam level elite female ice hockey athletes from youth ice hockey teams from Calgary and Edmonton (Canada) [c] cohort = Midget level elite female ice hockey athletes from youth ice hockey teams from Calgary and Edmonton (Canada), [d] calculated using raw data extracted, CI confidence interval, IR incidence rate, Level II a prospective cohort study or a randomised controlled trial
