# Supplementary material for: Incidence and Risk Factors for Sport-Related Concussion in Female Youth Athletes Participating in Contact and Collision Invasion Sports: A Systematic Review
Source: Sports Med. 2024 Dec 8;55(2):393–418. doi: 10.1007/s40279-024-02133-x (PMC11947075; doi:10.1007/s40279-024-02133-x)
Supplement: Supplementary file 9 — Supplementary file9 (PDF 335 KB) [file 40279_2024_2133_MOESM9_ESM.pdf]

Incidence and Risk Factors for Sport-Related Concussion in Female Youth Athletes Participating in Contact and Collision Invasion Sports: A Systematic Review

Sports Medicine

Laura Ernst[1], Jessica Farley[1], and Nikki Milne[1]

[1] Faculty of Health Science and Medicine, Bond University, Qld, Australia 4226

* Corresponding Author: Laura Ernst, Email: laura.ernst@student.bond.edu.au

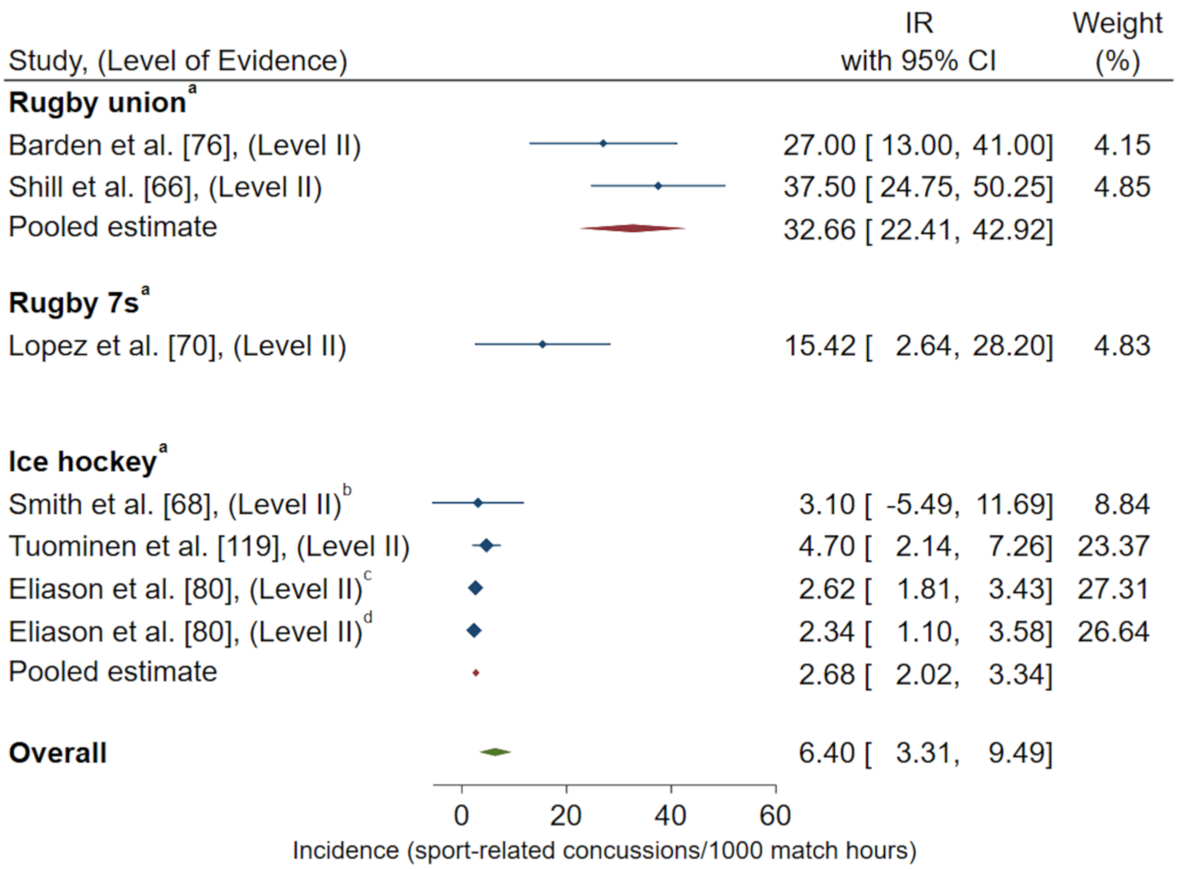

Online Resource 9. Pooled sport-related concussion incidence rate for collision invasion sports measured per 1000 match hours. Presented in chronological order according to publication year. [a] Collision sports, [b] Calculated using raw data extracted, [c] Under 15 female ice hockey athletes from youth ice hockey teams across British Columbia and Alberta (Canada), [d] Under 18 female ice hockey athletes from youth ice hockey teams across British Columbia and Alberta (Canada), CI confidence interval, IR incidence rate, Level II a prospective cohort study or a randomised controlled trial
