# Supplementary material for: Incidence and Risk Factors for Sport-Related Concussion in Female Youth Athletes Participating in Contact and Collision Invasion Sports: A Systematic Review
Source: Sports Med. 2024 Dec 8;55(2):393–418. doi: 10.1007/s40279-024-02133-x (PMC11947075; doi:10.1007/s40279-024-02133-x)
Supplement: Supplementary file 10 — Supplementary file10 (PDF 38 KB) [file 40279_2024_2133_MOESM10_ESM.pdf]

Incidence and Risk Factors for Sport-Related Concussion in Female Youth Athletes Participating in Contact and Collision Invasion Sports: A Systematic Review
Sports Medicine

Laura Ernst[1], Jessica Farley[1], and Nikki Milne[1]
[1] Faculty of Health Science and Medicine, Bond University, Qld, Australia 4226
* Corresponding Author: Laura Ernst, Email: laura.ernst@student.bond.edu.au

Online Resource 10. Non-modifiable sport-related concussion risk factors for all studies included in the systematic review

Risk factor	Study	Sport	Population compared	Sample size	No. of SRCs	Context	Statistical results	95% CI	p value
					**Non-Modifiable**				
					**No effect on risk**				
**Age**	Eliason et al. [77]	Ice hockey[b]	Ice hockey athletes in female only leagues (British Columbia and Alberta, Canada)	Under 15 = 245[a]<br>Under 18 = 138[a]	Under 15 = 38<br>Under 18 = 19	Match & practice	OR 1.15[a]	0.63-2.09[a]	0.646[a]
			Female ice hockey athletes in mixed sex leagues (British Columbia and Alberta, Canada)	Under 15 = 61[a]<br>Under 18 = 22[a]	Under 15 = 8<br>Under 18 = 4		OR 0.68[a]	0.18-2.53[a]	0.564[a]
	O'Kane et al. [104]	Soccer	Elite club athletes (Washington State, USA)	-	Under 14 = 19<br>Under 15 = 17	Match & practice	IRR 0.78[a]	0.38-1.59[a]	0.448[a]
	Schneider et al. [111]	Ice hockey[b]	Elite club athletes (Edmonton, Canada)	Bantam = 50<br>Midget = 69	Bantam (13-14 years) = 9<br>Midget (15-17 years) = 13	Match & practice	IRR 1.20[a]	0.45-3.02[a]	0.6741[a]

[a] calculated by the authors, [b] collision sports, - not reported, not investigated, or could not be calculated by the authors, IRR incidence rate ratio, OR odds ratio, SRC sport-related concussion, USA United States of America
