# Supplementary material for: Incidence and Risk Factors for Sport-Related Concussion in Female Youth Athletes Participating in Contact and Collision Invasion Sports: A Systematic Review
Source: Sports Med. 2024 Dec 8;55(2):393–418. doi: 10.1007/s40279-024-02133-x (PMC11947075; doi:10.1007/s40279-024-02133-x)
Supplement: Supplementary file 11 — Supplementary file11 (PDF 154 KB) [file 40279_2024_2133_MOESM11_ESM.pdf]

Incidence and Risk Factors for Sport-Related Concussion in Female Youth Athletes Participating in Contact and Collision Invasion Sports: A Systematic Review

Sports Medicine

Laura Ernst[1], Jessica Farley[1], and Nikki Milne[1]

[1] Faculty of Health Science and Medicine, Bond University, Qld, Australia 4226

* Corresponding Author: Laura Ernst, Email: laura.ernst@student.bond.edu.au

Online Resource 11. Potentially-modifiable sport-related concussion risk factors for all studies included in the systematic review

Risk factor	Study	Sport	Population compared	Sample size	No. of SRCs	Context	Statistical results	95% CI	p value
				**Potentially-modifiable**					
				**Increased risk**					
**Playing position**	Tuominen et al. [119]	Ice hockey[b]	World Championships tournament athletes	-	-	Centre vs defence in matches	OR 5.44	1.45-20.41	-
				**No effect on risk**					
	Lopez et al. [70]	Rugby 7s[b]	Rugby 7s tournaments athletes (USA)	-	Backs = 5[a] Forwards = 2[a]	Back vs forwards in matches	IRR 1.87[a]	0.31-19.67[a]	0.4847[a]
	Tuominen et al. [119]	Ice hockey[b]	World Championships tournament athletes	-	-	Wing vs defence in matches	OR 1.00	0.20-4.94	-
						Goalie vs defence in matched	OR 0.66	0.07-6.35	-
				**Increased risk**					
**Mechanism of injury**	Gessel et al. [90]	Basketball	High School athletes (USA)	-	-	A greater proportion of SRCs compared to all other injuries occurred whilst defending vs any other activity	IPR 1.56	1.50-1.62	<0.01

	Marar et al. [29]	Basketball	High school athletes (USA)	-	Proportion of SRCs sustained while defending = 29.8% Proportion of SRCs sustained in any other activity = 13.6%	SRCs whilst defending vs SRCs occurring from any other activity among females in matches & practice	IPR 2.20	1.60-3.10	<0.001
	Tuominen et al. [119]	Ice hockey[b]	World Championships tournament athletes	-	-	Checking vs other mechanisms	OR 5.35	1.87-15.29	-
				**No significant difference in risk**					
	Kerr et al. [95]	Soccer	High school athletes (USA)	-	Player to player contact = 385 All other mechanisms = 526	All other mechanisms vs player to player contact in match & practice	IRR 0.73[a]	0.64-0.84[a]	<0.0001[a]
					Contact with the ball = 336 All other mechanisms = 575	All other mechanisms vs contact with the ball in match & practice	IRR 0.58[a]	0.51-0.67[a]	<0.0001[a]
					Player to player contact = 385 Contact with the ball = 336	Contact with the ball vs player to player contact in match & practice	IRR 1.15[a]	0.99-1.33[a]	0.0681[a]
	Shill et al. [79]	Rugby union[b]	High school athletes (USA)	F = 361	Ball carrier = 19 Tackler = 30	Tackle related SRCs to ball carrier vs tackler in matches	IRR 0.63[a]	0.34 - 1.16[a]	0.119[a]
					Ball carrier = 7 Tackler = 4	Tackle related SRCs to ball	IRR 1.75[a]	0.45-8.15[a]	0.388[a]

						carrier vs tackler in practice			
					Ball carrier = 26<br>Tackler = 34	Tackle related SRCs to ball carrier vs tackler in match & practice combined	IRR 0.77[a]	0.44-1.31[a]	0.306[a]
	Tuominen et al. [119]	Ice hockey[b]	World Championships tournament athletes	-	-	Check to the head in matches vs other mechanisms	OR 0.54	0.07-4.09	-
						Checking from behind vs other mechanisms	OR 0.24	0.01-4.00	-
**No significant difference in risk**									
**Years of experience & mechanism of injury**	Shill et al. [79]	Rugby union[b]	High school athletes (USA)	F = 361	-	Level of experience (none vs 1 year) for tackle related SRC	IRR 1.15	0.69-1.19	-
					-	Level of experience (none vs 2+ years) for tackle related SRC	IRR 1.18	0.75-1.87	-
					-	Level of experience (none vs 1 year) for SRCs to tacklers during matches	IRR 0.72	0.25-2.06	-
					-	Level of experience (none vs 2+ years) for SRCs to tacklers during matches	IRR 1.45	0.60-3.50	-

					-	Level of experience (none vs 1 year) for SRCs to ball-carrier during matches	IRR 1.59	0.79-3.17	-
					-	Level of experience (none vs 2+ years) for SRCs to ball-carrier during matches	IRR 0.48	0.06-4.05	-
**Decreased risk**									
**Previous history of sports-related concussion**	Rauh et al. [108]	Basketball	High school athletes (USA)	-	Repeat SRC = 4[a] (1.7%)<br>New SRC = 54[a] (3.6%)	Repeat SRC vs new SRC in match & practice	IRR 0.07[a]<br>Ratio 0.47	0.02-0.20[a]	<0.0001[a]
		Field hockey		-	Repeat SRC = 3[a] (1.9%)<br>New SRC = 68[a] (2.4%)		IRR 0.04[a]<br>Ratio 0.77	0.01-0.14[a]	<0.0001[a]
		Soccer		-	Repeat SRC = 1[a] (1.6%)<br>New SRC = 11[a] (4.3%)		IRR 0.09[a]<br>Ratio 0.38	0.0-0.63[a]	0.0034[a]
**Increased risk**									
**Session time (period of practice or match)**	Covassin et al. [88]	Soccer	High school athletes (Michigan, USA)	-	-	Beginning vs middle of practice or match	RR 6.14	4.34-8.68	-
				-	-	Beginning vs end of practice or match	RR 3.84	2.68-5.51	-
				-	-		RR 1.60	1.30-2.00	-

		Basketball		-	-	Middle vs end of practice or match	RR 5.16	3.73-7.15	-
						Beginning vs middle of practice or match			-
				-	-	Beginning vs end of practice or match	RR 4.40	3.16-6.12	
**No effect on risk**									
	Covassin et al. [88]	Basketball	High school athletes (Michigan, USA)	-	-	Middle vs end of practice or match	RR 1.18	0.97-1.42	-
		Lacrosse		-	-	Beginning vs middle of practice or match	RR 2.40	0.85-6.80	-
				-	-	Beginning vs end of practice or match	RR2.60	0.93-7.28	-
				-	-	Middle vs end of practice or match	RR 0.92	0.42-2.02	-
**No effect on risk**									
**League type (female only league vs mixed sex league)**	Eliason et al. [77]	Ice hockey[b]	Ice hockey athletes in Under 15 age group leagues of all levels (British Columbia and Alberta, Canada)	F only league = 245[a] Mixed sex league = 61[a]	F only league = 38 Mixed sex league = 8	Competing in F only league vs mixed sex league	OR 1.22[a]	0.22-2.35[a]	0.64[a]
			Ice hockey athletes in Under 18 age group leagues of all levels (British Columbia and Alberta, Canada)	F only league = 138[a] Mixed sex league = 22[a]	F only league = 19 Mixed sex league = 4		OR 0.72[a]	0.22-2.35[a]	0.585[a]
**Higher risk in matches**									

**Session type (match vs practice)**	Castile et al. [115]	Soccer	High school athletes (USA)	-	Match = 178 Practice = 31	New SRC match vs practice	IRR 13.61[a]	-	-
				-	Match = 29 Practice = 4	Recurrent SRC match vs practice	IRR 17.02	5.98-48.42	<0.0001
				-	Match = 207 Practice = 35	Total SRC match vs practice	IRR 13.77[a]	-	-
		Basketball		-	Match = 120 Practice = 33	New SRC match vs practice	IRR 8.65[a]	-	-
				-	Match = 18 Practice = 9	Recurrent SRC match vs practice	IRR 4.73	2.13-10.53	-
				-	Match = 138 Practice = 42	Total SRC match vs practice	IRR 7.8[a]	-	-
	Clifton et al. [85]	Basketball	High school athletes (USA)	-	Match = 357 Practice = 122	Total SRC match vs practice	IRR 6.82[a]	5.54-8.45[a]	<0.0001[a]
	Comstock et al. [86]	Soccer	High school athletes (USA)	-	-	Heading related SRCs in match vs practice	IRR 15.10	9.70-24.50	-
	Comstock et al. [87]	Lacrosse	High school athletes (USA)	-	Match = 278 Practice = 1-6	Total SRC match vs practice	IRR 5.87	4.69-7.34	-
	DiStefano et al. [89]	Soccer	High school athletes (USA)	-	Match = 537 Practice = 90	Match vs practice	IRR 14.22[a]	-	-
	Haarbauer-Krupa et al. [91]	Soccer	High school athletes (USA)	-	Match = 439 Practice = 74	Match vs practice	IRR 13.40	10.50-17.20	-
		Basketball		-	Match = 287 Practice = 103		IRR 6.30	5.00-7.90	-
		Lacrosse		-	Match = 78 Practice = 29		IRR 5.80	3.80-9.00	-
		Field hockey		-	Match = 72 Practice = 32		IRR 4.80	3.20-7.30	-

	Herman et al. [92]	Lacrosse	High school athletes (USA)	-	Match = 89<br>Practice = 52	Match vs practice	IRR 4.44[a]	3.14-6.43[a]	<0.0001[a]
	Kerr et al. [96]	Soccer	High school athletes (USA)	-	Match = 864<br>Practice = 191	Match vs practice	IRR 10.17	8.70-11.90	-
		Basketball		-	Match = 504<br>Practice = 142		IRR 7.81	6.48-9.41	-
		Lacrosse		-	Match = 152<br>Practice = 57		IRR 6.03	4.45-8.18	-
		Field hockey		-	Match = 77<br>Practice = 22		IRR 7.49	4.66-12.03	-
	Kerr et al. [95]	Soccer	High school athletes (USA)	-	Match = 743<br>Practice = 168	Match vs practice	IRR 10.06	8.51-11.90	-
	Lynall et al. [116]	Field hockey	High school athletes (USA)	-	Match = 107<br>Practice = 45	Match vs practice	IRR 5.14[a]	3.60-7.46	<0.0001[a]
	Marar et al. [29]	Soccer	High school athletes (USA)	-	Match = 133<br>Practice = 26	Match vs practice	IRR 11.60	7.60-17.60	-
		Field hockey		-	Match = 29<br>Practice = 22		IRR 2.90	1.70-5.10	-
		Basketball		-	Match = 85<br>Practice = 22		IRR 9.20	5.50-14.20	-
		Lacrosse		-	Match = 45<br>Practice = 15		IRR 6.60	3.80-12.10	-
	O'Connor et al. [102]	Basketball	High school athletes (USA)	-	Match = 81<br>Practice = 47	Match vs practice	IRR 4.74	3.31-6.79	-
		Soccer		-	Match = 66<br>Practice = 40		IRR 5.80	3.91-8.58	-
		Lacrosse		-	Match = 30<br>Practice = 26		IRR 3.42	2.02-5.78	-
		Field hockey		-	Match = 39<br>Practice = 27		IRR 3.99	2.44-6.51	-

	Pierpoint et al. [106]	Lacrosse	High school athletes (USA)	-	Match = 125<br>Practice = 52	Match vs practice	IRR 5.33[a]	3.83-7.51[a]	<0.0001[a]
	Powell and Barber-Foss [117]	Soccer	High school athletes (USA)	-	-	Match vs practice	IRR 14.40	9.00-23.00	-
		Basketball		-	-		IRR 6.10	3.80-9.70	-
		Field hockey		-	-		IRR 14.40	4.60-44.90	-
	Rechel et al. [109]	Basketball	High school athletes (USA)	-	Match = 36[a]<br>Practice = 6[a]	Match vs practice	IRR 14.95[a]	6.24-43.3[a]	<0.0001[a]
							IPR 5.83	2.05-16.49	-
		Soccer		-	Match = 42[a]<br>Practice = 10[a]		IRR 9.50[a]	4.69-21.23[a]	<0.0001[a]
							IPR 1.93	0.85-4.41	-
	Rivara et al. [67]	Soccer	High school athletes (Washington State, USA)	F = 288	Match = 27<br>Practice = 5	Match vs practice	IRR 12.40	4.70-41.30	<0.0001[a]
	Shill et al. [66]	Rugby union[b]	High school athletes (USA)	F = 361	Match = 62<br>Practice = 16	Match vs practice	IRR 37.62[a]	21.45-69.85[a]	<0.0001[a]
	Warner et al. [113]	Lacrosse	High school athletes (USA)	-	Match = 194<br>Practice = 79	Match vs practice	IRR 5.50	4.20-7.10	-
	Xiang et al. [114]	Lacrosse	High school athletes (USA)	-	Match = 72[a]<br>Practice = 33[a]	Match vs practice	IRR 4.89	3.24-7.39	-
**No effect on risk**									
	Baron et al. [82]	Lacrosse	High school athletes (New York, USA)	HG cohort n = 1585	Match = 1<br>Practice = 1	Match vs practice	IRR 1.931[a]	0.025-151/59[a]	0.6823[a]
	Le Gall et al. [107]	Soccer	Elite club athletes	F = 119	Match = 1<br>Practice = 1	Match vs practice	IRR 0.84[a]	0.11-701.46[a]	0.2103[a]
**Higher rate of SRC with access to medical staff**									
**Access to medical staff**	Pierpoint et al. [69]	Soccer	High school athletes (Chicago, USA)	-	Access to AT = 148<br>No access to AT = 2	Access to AT in match & practice vs no access to	IRR 8.05	2.00-32.51	<0.05
		Basketball		-	Access to AT = 109<br>No access to AT = 3	AT in match & practice	IRR 4.50	1.43-14.16	<0.05

					**No effect on risk**				
	Kroshus et al. [98]	Soccer	High school athletes (Washington, USA)	-	-	Access to athletic trainer vs no access to athletic trainer (0-1 vs 2-5 SRCs diagnosed)	RR Model 1 (unadjusted) 0.9	Model 1 0.80-2.20	-
							RR Model 2 (adjusted for school location) 0.9	Model 2 0.50-1.50	-
							RR Model 3 (adjusted for school enrolment) 1.0	Model 3 0.60-1.80	-
							RR Model 4 (adjusted for school lunch eligibility) 1.1	Model 4 0.60-2.00	-
				-	-	Access to athletic trainer vs no access to athletic trainer (0-1 vs 5+ SRCs diagnosed)	RR Model 1 (unadjusted) 1.5	Model 1 0.60-3.70	-
							RR Model 2 (adjusted for school location) 1.0	Model 2 0.30-3.10	-
							RR Model 3 (adjusted for school enrolment) 1.0	Model 3 0.30-2.70	-
							RR Model 4 (adjusted for school lunch eligibility) 1.2	Model 4 0.40-3.50	-

[a] calculated by the authors, [b] collision sports, - not reported, not investigated, or could not be calculated by the authors, AR attributable risk, AT athletic trainer, F female, IPR injury proportion ratio, IRR incidence rate ratio, OR odds ratio, RR risk ratio, SRC sport-related concussion, USA United States of America
