# Supplementary material for: Incidence and Risk Factors for Sport-Related Concussion in Female Youth Athletes Participating in Contact and Collision Invasion Sports: A Systematic Review
Source: Sports Med. 2024 Dec 8;55(2):393–418. doi: 10.1007/s40279-024-02133-x (PMC11947075; doi:10.1007/s40279-024-02133-x)
Supplement: Supplementary file 12 — Supplementary file12 (PDF 66 KB) [file 40279_2024_2133_MOESM12_ESM.pdf]

Incidence and Risk Factors for Sport-Related Concussion in Female Youth Athletes Participating in Contact and Collision Invasion Sports: A Systematic Review

Sports Medicine

Laura Ernst[1], Jessica Farley[1], and Nikki Milne[1]

[1] Faculty of Health Science and Medicine, Bond University, Qld, Australia 4226

* Corresponding Author: Laura Ernst, Email: laura.ernst@student.bond.edu.au

Online Resource 12. Modifiable sport-related concussion risk factors for all studies included in the systematic review

Risk factor	Study	Sport	Population compared	Sample size	No. of SRCs	Context	Statistical results	95% CI	p value
					**Modifiable**				
					**SRC risk decreased with increased neck strength**				
**Neck strength**	Collins et al. [73]	Soccer, basketball and lacrosse combined	High school athletes (USA)	Total sample = 3002	Total SRC = 107<br>Total no SRC = 2895	Comparison of neck strength measurements between female athletes who sustained a concussion and those who did not	For every one-pound increase in neck strength, odds of concussion decreased by 5%, OR = 0.95	0.92-0.98	-
					Overall neck strength<br>SRC = 107<br>No SRC = 2885		-	-	0.052
					Neck extension<br>SRC = 107<br>No SRC = 2878		-	-	0.061
					Neck flexion<br>SRC = 107<br>No SRC = 2883		-	-	0.050
					Right lateral<br>SRC = 107<br>No SRC = 2885		-	-	0.053
					Left lateral<br>SRC = 107<br>No SRC = 2884		-	-	0.078

**Decreased risk of SRC with protective equipment**									
**Protective equipment**	Baron et al. [82]	Lacrosse	High school athletes (USA)	HG cohort n = 1585<br>Control cohort = -	HG = 1[a]<br>No HG = 182[a]	HG vs no HG in matches	IRR 0.15	0.00-0.86	0.023
					HG = 2[a]<br>No HG = 256[a]	HG vs no HG in match & practice combined	IRR 0.24	0.03-0.87	0.022
	Herman et al. [92]	Lacrosse	High school athletes (USA)	-	No HG = 74<br>HG = 15	No HG vs HG in matches	IRR 1.74	1.00-3.02	-
				-	No HG = 116<br>HG = 25	No HG vs HG in match & practice combined	IRR 1.59	1.03-2.45	-
						HG vs no HG in match & practice combined	IRR 0.63[a]	0.39-0.98[a]	0.03[a]
**Protective equipment has no effect on risk**									
	Baron et al. [82]	Lacrosse	High school athletes (USA)	HG cohort n = 1585<br>Control cohort = -	HG = 1[a]<br>No HG = 74[a]	HG vs no HG in practice	IRR 0.43	0.01-2.48	0.663
	Herman et al. [92]	Lacrosse	High school athletes (USA)	-	No HG = 42<br>HG = 10	No HG vs HG in practice	IRR 1.42	0.71-2.83	-
	Kriz et al. [72]	Field hockey	High school athletes (USA)	-	MPE = 18<br>No MPE = 75	MPE vs no MPE	IRR 0.95[a]	0.54-1.61[a]	0.8762[a]
	Kriz et al. [71]	Field hockey	High school athletes (USA)	-	MPE = 134<br>No MPE = 75	MPE vs no MPE	IRR 1.30[a]<br><br>OR 0.74	0.97-1.75[a]<br><br>0.58-1.02	0.0660[a]<br><br>0.068
	McGuine et al. [103]	Soccer	High school athletes (USA)	Intent-to-treat no HG = 999<br>Intent-to-treat HG = 1031	No HG = 60<br>HG = 48	HG vs no HG in match & practice	RR 0.90<br><br>HR 0.86	0.55-1.48<br><br>0.54-1.36	0.683<br><br>0.520
				As-treated no HG = 998	No HG = 65<br>HG = 43		RR 0.64	0.38-1.08	0.094

				As-treated HG = 1032			HR 0.70	0.43-1.15	0.154
**Increased rate of SRC post TBI laws**									
**Laws, rules or governance**	Schallmo et al. [110]	Soccer	High school athletes (USA)	-	-	Post TBI law vs pre TBI law in match & practice	IRR 2.52	1.83-3.49	<0.01
							IPR 2.27	2.24-2.30	<0.01
		Basketball		-	-		IRR 1.79	1.22-2.62	<0.01
							IPR 2.15	2.11-2.20	<0.01
**No effect on risk**									
	O'Kane et al. [65]	Soccer	Elite club athletes (Washington State, USA)	-	-	After Lystedt Law vs before Lystedt Law in Under 14 teams	RR 0.60	0.20-2.10	-
						After Lystedt Law vs before Lystedt Law in Under 15 teams	RR 1.70	0.50-9.60	-
	Smith et al. [68]	Ice hockey[b]	Ice hockey tournaments athletes (Minnesota, USA)	IFP = 171 NIFP = 157	IFP group = 1 NIFP group = 0	IFP vs NIFP in matches	RR 0.42 *(analysed using a penalised likelihood approach in logistic regression)*	0.02-10.34	0.596

[a] calculated by the authors, [b] collision sports, - not reported, not investigated, or could not be calculated by the authors, AR attributable risk, HG head-gear, HR hazard ration, IFP intensified fair play, IPR injury proportion ratio, IRR incidence rate ratio, MPE mandated protective eyewear, NIFP non-intensified fair play, OR odds ratio, RR risk ratio, SRC sport-related concussion, TBI traumatic brain injury, USA United States of America
